# Impact of tree-based interventions in addressing health and wellbeing outcomes in rural low-income and middle-income settings: a systematic review and meta-analysis

**DOI:** 10.1016/S2542-5196(25)00003-8

**Published:** 2025-02-01

**Authors:** Peninah Murage, Blanca Anton, Faraja Chiwanga, Roberto Picetti, Tabby Njunge, Syreen Hassan, Sarah Whitmee, Jane Falconer, Hugh Sharma Waddington, Rosemary Green

**Affiliations:** Department of Public Health, Environments and Society, London School of Hygiene & Tropical Medicine, London, UK; Department of Population Health, London School of Hygiene & Tropical Medicine, London, UK; Muhimbili National Hospital, Dar es Salaam, Tanzania; LEAD (Leadership Formation, Environmental Conservation and Action for Development) Foundation, Dodoma, Tanzania; Department of Population Health, London School of Hygiene & Tropical Medicine, London, UK; Conservation International, Nairobi, Kenya; Department of Non-communicable Disease Epidemiology, London School of Hygiene & Tropical Medicine, London, UK; Department of Population Health, London School of Hygiene & Tropical Medicine, London, UK; Library Archive & Open Research Services, London School of Hygiene & Tropical Medicine, London, UK; Department of Population Health, London School of Hygiene & Tropical Medicine, London, UK

## Abstract

The impact of nature-based solutions on human health is increasingly recognised; however, our understanding of the strength of evidence and the extent to which it supports policy and practice is insufficient. We aimed to assess the health and wellbeing impacts of solutions in low-income and middle-income settings in which trees are a central feature in the protection, restoration, and sustainable management of landscapes. For this systematic review and meta-analysis, we searched Web of Science, Embase, APA PsycInfo, MEDLINE ALL, Global Health, Global Index Medicus, GreenFILE, SciELO, EconLit, and Africa-Wide Information for studies that evaluated the impacts of relevant interventions on health and wellbeing. Searches were limited to records published from Jan 1, 2000, to the search date; an initial search was conducted on Nov 23, 2021, and was updated on Feb 27–28, 2023. We extracted data from studies comparing interventions with matched controls, calculated standardised mean differences, and pooled the effects using random-effects meta-analysis with adjustments for potential effect dependence. Studies were assessed for quality using seven risk-of-bias domains. Our search identified 23 402 studies, of which 54 were included in the meta-analysis. We found significant positive pooled effects for agricultural yields (standardised mean difference 0·41 [95% CI 0·11 to 0·70]), dietary diversity (0·10 [0·02 to 0·18]), total household income (0·21 [0·09 to 0·33]), poverty reduction (0·17 [0·07 to 0·27]), child growth (0·11 [0·00 to 0·22]), and self-reported wellbeing (0·21 [0·00 to 0·43]). Loss of income from timber production could be a negative outcome (−0·13 [−0·29 to 0·02]); however, these effects might be partially offset by increased income from non-timber forest products (0·32 [0·04 to 0·61]). Effects varied substantially by intervention type, with more positive effects associated with interventions in which the primary target was livelihood improvement than with interventions that targeted biodiversity or carbon mitigation. However, cautious interpretation is urged owing to the low certainty of the evidence. In conclusion, evidence suggests that tree-based solutions can support the health and wellbeing of the implementing communities. Such evidence strengthens the case for aligning health objectives with the goals of nature-based solutions by making community wellbeing an integral component of conservation programmes. Future studies should examine a wider range of outcomes that have direct relevance for health.

## Introduction

Ecosystem degradation, human health, and socio-economic development are deeply intertwined. Improvements in human health, such as longer life expectancies, can be linked to our exploitation of the environment for natural services and resources.1 However, the depletion of natural resources and the erosion of natural systems are already having harmful impacts on human health and wellbeing^1^ and undermining progress towards the UN’s Sustainable Development Goals. Extreme heat events and changes in precipitation patterns attributed to climate change over the past decades are striking examples of how shifts in natural systems can devastate human health—increasing heat stress, compromising food and nutritional security, and leading to loss of life and property.^2^ Human health has the opportunity to thrive when natural systems Flourish^3^ and access to resources to support livelihoods and wellbeing is maintained. Nature-based solutions or actions that protect, sustainably manage, and restore natural and modified ecosystems^4^ have the potential to address multiple societal challenges, including adapting to and mitigating climate change, protecting and restoring biodiversity, and safeguarding livelihoods.^4^ As central features in many terrestrial and coastal habitats, trees are often a key component of nature-based solutions.^5^ Trees are fundamental to ecosystem services that support life on Earth, through the supply of food to support nutrition and natural resources for building and energy; the provision of recreational spaces to support mental and physical health; and the regulation of ecosystem processes (by improving air and water quality), climate and natural hazards (by providing a buffer against extreme events), and pathogen and pests (by affecting the abundance of disease vectors).^3^

Many nature-based solutions are primarily implemented as actions to mitigate climate change, actions to reverse ecological degradation, or both. Early interventions were criticised for giving little consideration to their impact on human wellbeing, which carried the risk of unintended trade-offs, poor local adoption and, in extreme cases, erosion of the rights of Indigenous people, for example by limiting access to forest resources or land for cultivation.^6–8^ In low-income and middle-income countries (LMICs) where agricultural production is the main income source, there could be large trade-offs between socioeconomic objectives on poverty alleviation and environmental goals such as biodiversity conservation from poorly designed nature-based solutions.^7,9–12^ Due consideration must therefore be given to the societal co-benefits of nature-based solutions and to reducing or compensating for negative impacts, which can be achieved only through ensuring full participation of local communities and protecting their ecological and cultural rights.^13,14^

Tree-based solutions include tree intercropping,^15–17^ establishing or strengthening protected areas,^18^ payments for ecosystem services (PES),^19,20^ reducing emissions from deforestation and forest degradation in developing countries (REDD+),^21^ and restoration of mangrove ecosystems along coastlines. These interventions are being implemented at a rapid pace, but evidence quantifying their impact and characterising how they work to deliver the anticipated outcomes or generate trade-offs is scarce.^22^ The evidence gaps result from methodological challenges in measuring attribution (eg, as a result of difficulties in untangling complex feedback loops between conservation interventions and socioecological systems^23^) and the spatial–temporal differences in environmental exposures and health outcomes leading to datasets that are not easy to link and compare.^11^ Well designed impact-evaluation studies that compare outcomes in communities implementing the solutions to matched controls can provide the methodological rigour required to address the deficit in quantitative evidence.^11,22,24^ However, complementary qualitative approaches, such as process evaluations are necessary to help to unravel contextual mechanisms that could explain how the intervention works to deliver the intended outcomes or to generate unintended impact.

Evidence regarding our understanding of the impacts of interventions on human health and wellbeing is particularly scarce. In LMIC settings, this lack of evidence has implications for the scalability of interventions; for example, agroforestry is widely promoted as a development tool to meet Sustainable Development Goals, but major evidence gaps remain in demonstrating its impact.^25–27^ A 2022 evidence map on the ecological, economic, and social outcomes of agroforestry solutions found 64 existing reviews; however, only six reported social outcomes.^26^ This low proportion highlights a profound imbalance in which environmental outcomes have been synthesised intensively but social outcomes have hardly been considered, limiting general conclusions about the social, health, and wellbeing outcomes of agroforestry. Likewise, existing studies—including a meta-analysis of agroforestry solutions^25^ and syntheses of PES solutions^28^ and REDD+^29^—focused on individual terrestrial interventions and did not consider coastal interventions such as mangroves. Many tree-based conservation solutions have multiple complementary practices^10^ (appendix p 2) that hinder a single-intervention synthesis and warrant a broader synthesis to unpick contextual factors that can facilitate or impede impact. Many of the existing reviews are also outdated; a newer evidence synthesis is needed given the exponential growth in the literature on nature-based solutions in recent years.

Our systematic review and quantitative meta-analysis is, to our knowledge, the first comprehensive synthesis of health, wellbeing, and societal outcomes of tree-based solutions implemented in rural landscapes across LMICs. Ongoing ecosystem degradation across these settings has a disproportionate impact on marginalised communities who are heavily reliant on fragile ecosystems for sustenance^30^ and have some of the lowest levels of wellbeing.^3^ These communities stand to benefit from well implemented solutions, but the risk of unintentional trade-offs would severely compromise any gains. The theory of change (appendix p 1) causally links inputs, processes, and ecosystem services to the desired outcomes in health and wellbeing that could be attributed to well implemented interventions.

## Methods

### Search strategy and selection criteria

This systematic review and meta-analysis aims to synthesise the existing evidence on the health and socioeconomic impacts of tree-based solutions imple-mented in rural LMIC settings, including interventions such as REDD+, agroforestry, PES, mangroves and coastal interventions, and other community forest initiatives that work through the protection, restoration, or sustainable management of trees in landscapes (appendix p 2).

The search strategy was developed using the population, intervention, comparator, and outcomes framework. We aimed to include people living in LMICs (as per the Organisation for Economic Co-operation and Development classification) and consider interventions—evaluated in a real-world setting—that adhered to the principles of nature-based solutions in terms of enhancing natural ecosystem processes and biodiversity, safeguarding local rights, and addressing societal challenges^4^ (appendix pp 3–6). Interventions that involved monoculture tree plantations and those with a focus on timber production and logging were excluded. Comparator populations were defined as either pre-intervention baselines or control sites where the intervention did not take place.

We considered a broad set of outcomes aligned with health, wellbeing, and socioeconomic status. Health and wellbeing outcomes were those related to food and nutrition, child growth (height-for-age, weight-for-age, and height-for-weight), water and sanitation, infectious diseases, air quality, thermal comfort, physical and mental health, maternal health, injuries, and non-communicable diseases, whereas socioeconomic outcomes were crop yields, poverty, income, employment, wealth, school attendance, gender equality, conflict, disaster risk reduction, labour productivity, and social cohesion. Studies were included only if they used experimental or quasi-experimental study designs to evaluate the effects of interventions on these outcomes.^28^

We excluded studies that used only qualitative or other observational methods with no comparator group; studies based in urban areas or those with no clearly defined tree-based intervention; and studies that did not examine health, wellbeing, or both, or socioeconomic outcomes. We also excluded systematic reviews and results published in non-peer-reviewed sources.

We searched for primary studies in the following databases: Web of Science (Science Citation Index Expanded, Social Sciences Citation Index, and Emerging Sources Citation Index), OvidSP Embase, OvidSP APA PsycInfo, OvidSP MEDLINE ALL, OvidSP Global Health, WHO Global Index Medicus, EBSCOhost GreenFILE, Web of Science SciELO, OvidSP EconLit, and EBSCOhost Africa-Wide Information. A database search was conducted on Nov 23, 2021, and updated on Feb 27–28, 2023. The results were limited to those published between Jan 1, 2000, and the search date. The search was not limited by language or study method. Search terms were selected after a review of titles and keywords in the known eligible literature. The Web of Science search is provided as an example in the appendix (pp 3–6); the complete search across all other databases can be found in Falconer and Murage (2024).^31^ Reference lists of identified systematic reviews were also scanned for additional relevant studies.

All records were exported to data-management software (EPPI Reviewer 6; EPPI Centre at the Social Science Research Unit of the UCL Institute of Education, University of London, London, UK) and duplicate records were removed using a built-in tool. For title and abstract screening, we used Priority Screening, an algorithm text-mining tool within the software that minimises screening burden and improves efficiency for large reviews.^32^ Priority Screening learns the characteristics of the included and excluded studies and uses this information to predict whether a given record is likely to be relevant or irrelevant^32^ (appendix p 7); the software then presents the records in order of probability of meeting the inclusion criteria, grouped into bands of 1–10 (or any other relevant banding specified by the user). This process is iterative and includes training the software to learn the inclusion criteria (by manually screening 10% of the references); running the software to screen the remaining references and presenting the records in bands (band 1 containing the most relevant records and band 10 containing the least relevant); and reviewing the most relevant bands to ensure that the most relevant studies are captured. We repeated these three steps until the point of saturation, at which bands 1–3 contained no relevant studies. Finally, we conducted a rapid manual title scan of all excluded studies in bands 1–10 to ensure that no relevant records were omitted. The screening process was done by four pairs of reviewers (PM and BA, FC and SH, TN and RG, and JF and RP) and discrepancies were resolved by a third reviewer (PM, TN, or FC). The manual title scan of the excluded studies was done by one reviewer (PM). To ensure inter-reviewer reliability,^32^ we established a baseline inclusion rate in the first step to estimate the likely number of relevant studies. If the abstract lacked sufficient detail to assess eligibility, the record was included and was passed on to full-text screening.

The study protocol was registered on PROSPERO (CRD42021291622); the only deviation from this protocol was that we included only studies that used experimental or quasi-experimental designs and excluded observational, cross-sectional, and modelling studies. The study follows PRISMA guidelines (appendix pp 8–9).^33^

### Data analysis and synthesis

Articles that met the inclusion criteria after full-text screening were assessed for risk of bias and data on the following were extracted: author, year of publication, brief objective of the intervention and practices promoted, year of implementation, location, study design and methods, unit of data collection and analysis, type of outcome evaluated, sample size of treatment and control groups, effect sizes, standard errors, p value, and t value. Three reviewers (PM, BA, and FC) did the quality assessment and data extraction, and a fourth reviewer (HSW) checked them for agreement. The four reviewers met regularly throughout the data-extraction stage to discuss any issues and ensure consistency in their approach.

A risk-of-bias assessment was conducted using a critical appraisal tool^34^ for randomised and non-randomised studies of effects developed by HSW. This tool is an adapted version of the Cochrane tool and assesses several types of bias: biases in confounding, selection, attrition, motivation, and performance; measurement and reporting errors; and analysis reporting biases (appendix pp 10–11).

In the meta-analyses, data extracted from the included studies were used to compare the estimated effect of the intervention on outcomes. For each study, we extracted the mean differences in outcomes in the intervention and control groups and calculated the standardised mean difference (SMD) effect sizes. For studies reporting both the mean difference between treated and control (X_t_–X_c_) populations and the standard error (SE), we calculated the effect size using the formula SMD=(X_t_–X_c_)/SD, where standard deviation, SD, was either reported in the paper or, where it was not reported, was calculated as:

$$\text{SD}=\frac{\text{SE}}{\sqrt{\left(\frac{1}{{\text{n}}_{\text{t}}}+\frac{1}{{\text{n}}_{\text{c}}}\right)}}$$

For studies reporting mean differences and p values only, we estimated the t value and standard error as t value=TINV (p value, (N)) and SE=X_t_–X_c_/t value.

The variance, V(SMD), was estimated as

$$\text{V}(\text{SMD})=\frac{\text{N}}{{\text{n}}_{\text{t}}\times {\text{n}}_{\text{c}}}+\frac{\text{SM}{\text{D}}^{2}}{2\text{N}}$$

and

$$\text{SE}(\text{SMD})=\sqrt{\text{V}(\text{SMD})}$$

For studies that generated effect sizes using multiple matching techniques or across multiple timepoints, we calculated weighted synthetic averages. Where possible, inferences from difference-in-difference estimation were selected for effect size data extraction in preference to other measures (eg, post-test measurement only). Effect sizes reported across multiple unrelated intervention locations were treated as independent. In the sensitivity analysis, three-level meta-analysis models were used to adjust for potential effect dependence in multiple outcomes arising from different samples but from the same study^35^ and to account for heterogeneity between and within studies.^35^ Studies generating effect sizes for multiple related constructs were discussed and the outcome that had the strongest alignment with the given health measure was selected.

When data allowed, we used meta-analysis to pool the effect sizes of the impacts of the interventions on agricultural yield, food security, dietary diversity, poverty reduction, self-reported wellbeing, income, child growth, and access to education. Relationships were considered appropriate for meta-analysis if three or more studies reported an equivalent outcome and if effect sizes could be generated and pooled. We used the metafor package in R (version 4.2.0) to conduct a random-effects meta-analysis and create forest plots with effect sizes from each included study. For each outcome, we report the pooled effects in forest plots as SMD (95% CI). Effect size magnitudes were designated as small (0·15 to <0·35), moderate (0·35 to <0·65), and large (≥0·65), using the thresholds recommended for related health outcomes.^36^

We used the *I* statistic (*I*^2^) to report the percentage of variance due to heterogeneity and reported the variability of the true effects using the tau-squared (τ^2^) statistic and Cochran’s Q statistic. We assessed potential publication bias due to missing evidence through funnel plots and used Egger’s regression to test for funnel plot asymmetry if at least ten studies were included in the meta-analysis. Our certainty in the body of evidence—overall and at the level of intervention groups—was assessed on the basis of the pooled effect size, 95% CI, number of studies that presented the outcome, and heterogeneity statistics. For outcomes with insufficient data to conduct meta-analyses or if outcome measures were not comparable, we provided a narrative discussion.

### Role of the funding source

The funders of the study had no role in study design, data collection, data analysis, data interpretation, or writing of the report.

## Results

**Figure 1:**
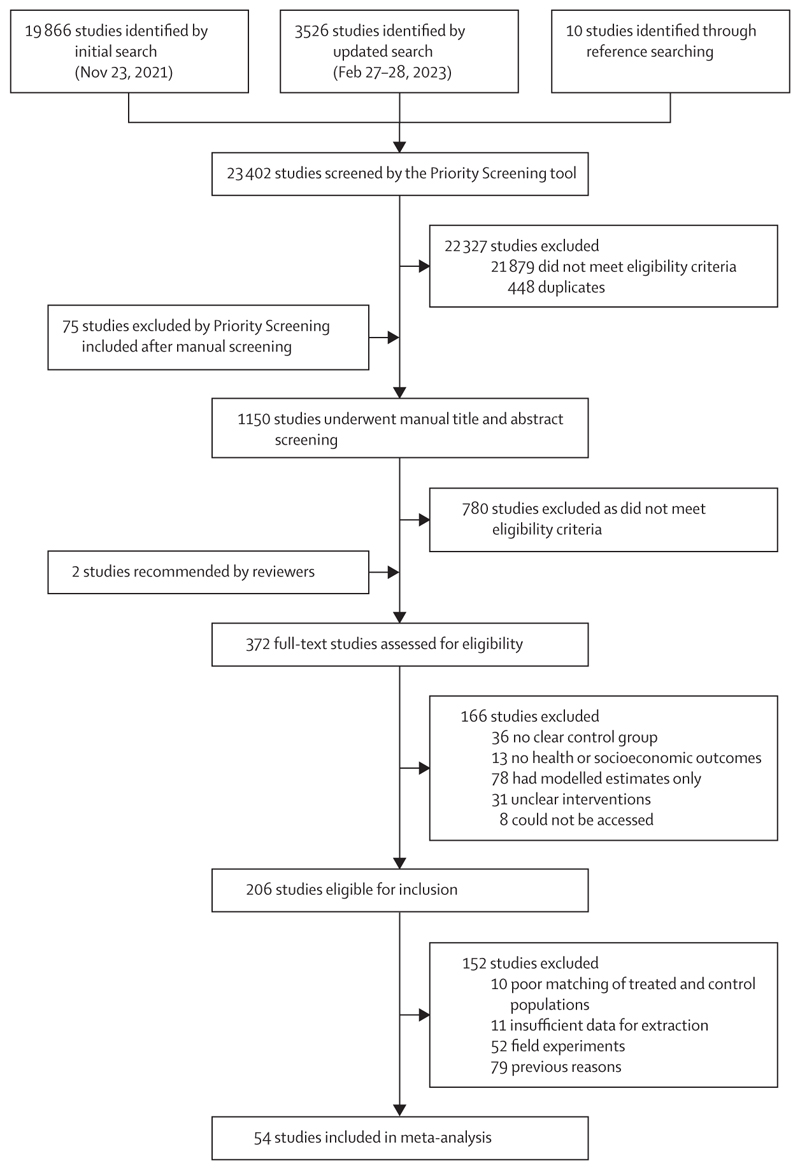
Study selection

**Figure 2:**
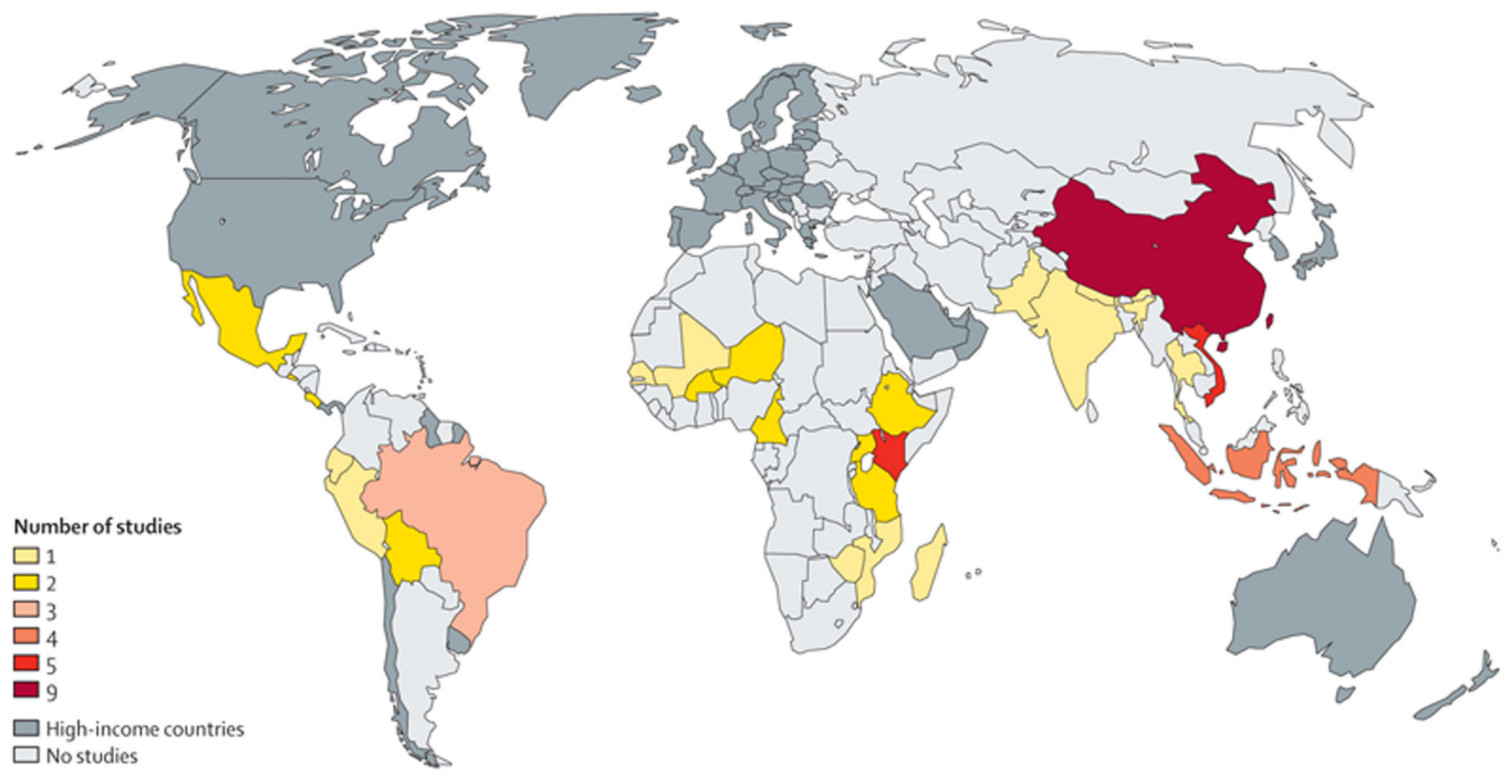
Distribution of included studies

Our initial search identified 23 402 studies, of which 372 underwent full-text review and 54 were included in the meta-analysis (figure 1, appendix pp 12–15). The studies were distributed across most regions (figure 2): the largest number of studies came from China (n=9), whereas Kenya and Viet Nam had five studies each.

The outcomes were grouped into three categories: food and nutrition (agricultural yields, food security, and dietary diversity); poverty and income (total household income and income from agricultural products, timber forest products, and non-timber forest products); and wellbeing and development (access to education, self-reported wellbeing, and child growth as measured by height-for-age, weight-for-age, and weight-for-height).

Tree-based interventions were thematically categorised into the following broad groups: REDD+,^21^ trees in croplands,^15–17^ protected areas,^18^ PES,^19,20^ collaborative forest management,^37^ mangroves, and the national forest protection programme.^38^ Interventions varied by biome type, but most major biomes were represented. These categories were based on how the intervention was defined in the study, although there were some overlaps across the groups in terms of the nature of implementation—eg, some PES schemes applied agroforestry techniques of integrating trees in croplands (see appendix p 2 for more detail on overlaps). There were also distinct differences in the intervention categories in terms of their overall objectives; eg, the primary aim of REDD+ is to enhance the removal of greenhouse gases, whereas integrating trees in croplands has a strong emphasis on livelihood improvement.

A substantial proportion of the included studies had a high risk of bias overall; a smaller proportion had a critical risk of bias (appendix pp 10–11) because appropriate techniques to minimise confounding bias were not used. Several studies were also classed as having performance bias because contamination between intervention and control groups was not adequately addressed (appendix pp 10–11).

For each outcome, we report the effect sizes (SMD [95% CI], p value) of the three-level meta-analytical model (adjusted for dependence), *I*^2^, and τ^2^, and also fit an effect moderator to show subgroup analyses by type of intervention. In the multilevel models, the metafor function in R estimates heterogeneity between the studies and within the study (for separate samples); we report this total heterogeneity alongside the pooled effect. The results of the unadjusted models (without adjustment for dependence) are shown in the appendix (p 16).

**Figure 3:**
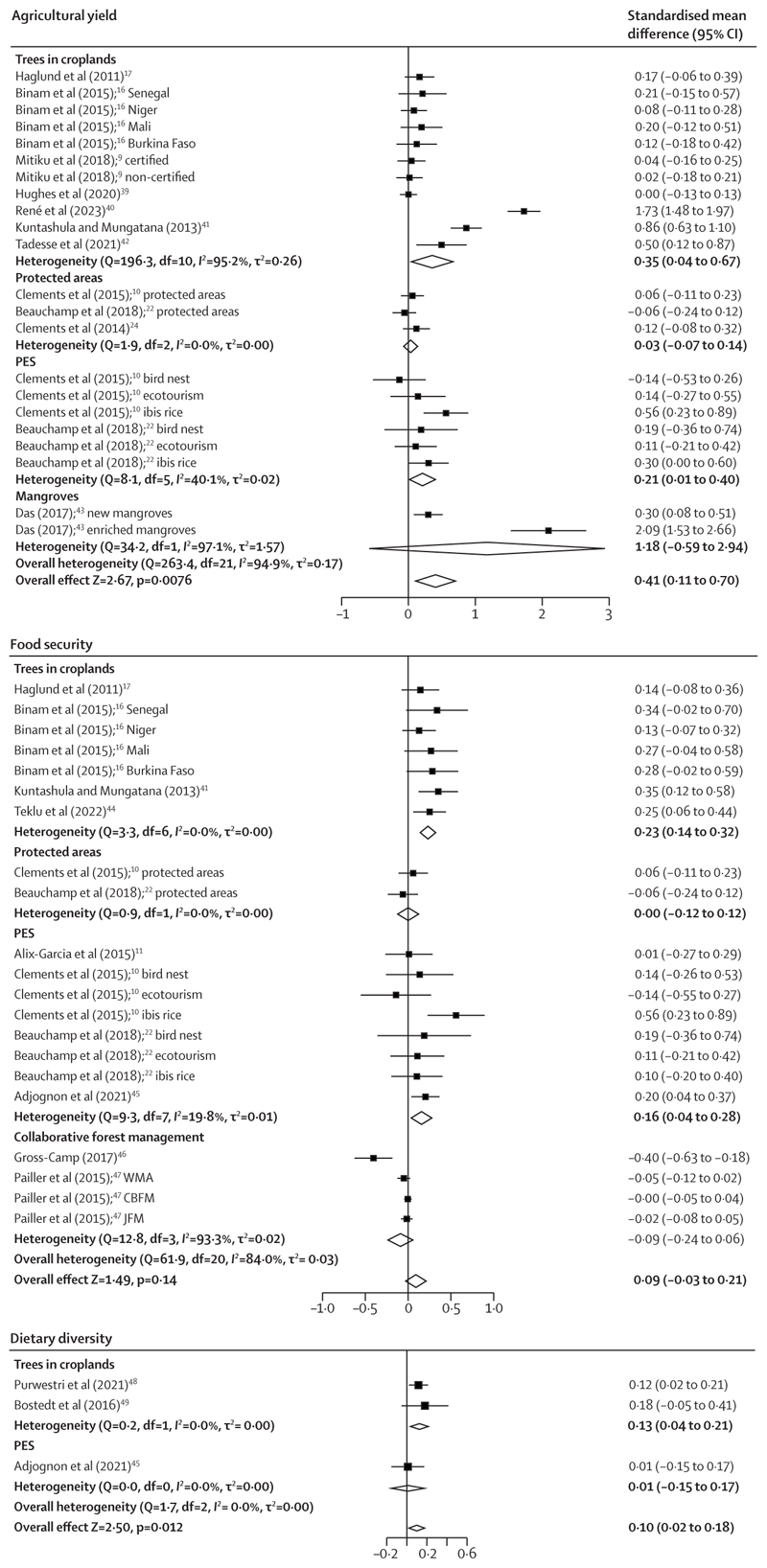
Forest plots of pooled and subgroup analysis effects of the impact of interventions on food and nutritional outcomes Positive effects indicate good outcomes. CBFM=community-based forest management. JFM=joint forest management. PES=payments for ecosystem services. WMA=wildlife management areas.

For outcomes related to food and nutrition, the impacts of interventions on agricultural yields and food security were examined in ten studies (more than 20 different analyses; figure 3). The pooled effect across all interventions showed a significant, moderate-sized positive impact on agricultural yield (SMD 0·41 [95% CI 0·11 to 0·70], p=0·0076; *I*^2^=94·9% and τ^2^=0·17; figure 3). The subgroup analysis found that interventions related to trees in croplands had the greatest significant effects (0·35 [0·04 to 0·67]; p=0·025; *I*^2^=95·2% and τ^2^=0·26). Smaller positive effects were found on dietary diversity (0·10 [0·02 to 0·18]; p=0·012; *I*^2^=0·0% and τ^2^=0·00), pooled from three studies. The effect on food security varied by type of intervention: trees in croplands and PES had significant positive effects, whereas other interventions such as protected areas and collaborative forest management had insignificant effects (figure 3).

**Figure 4:**
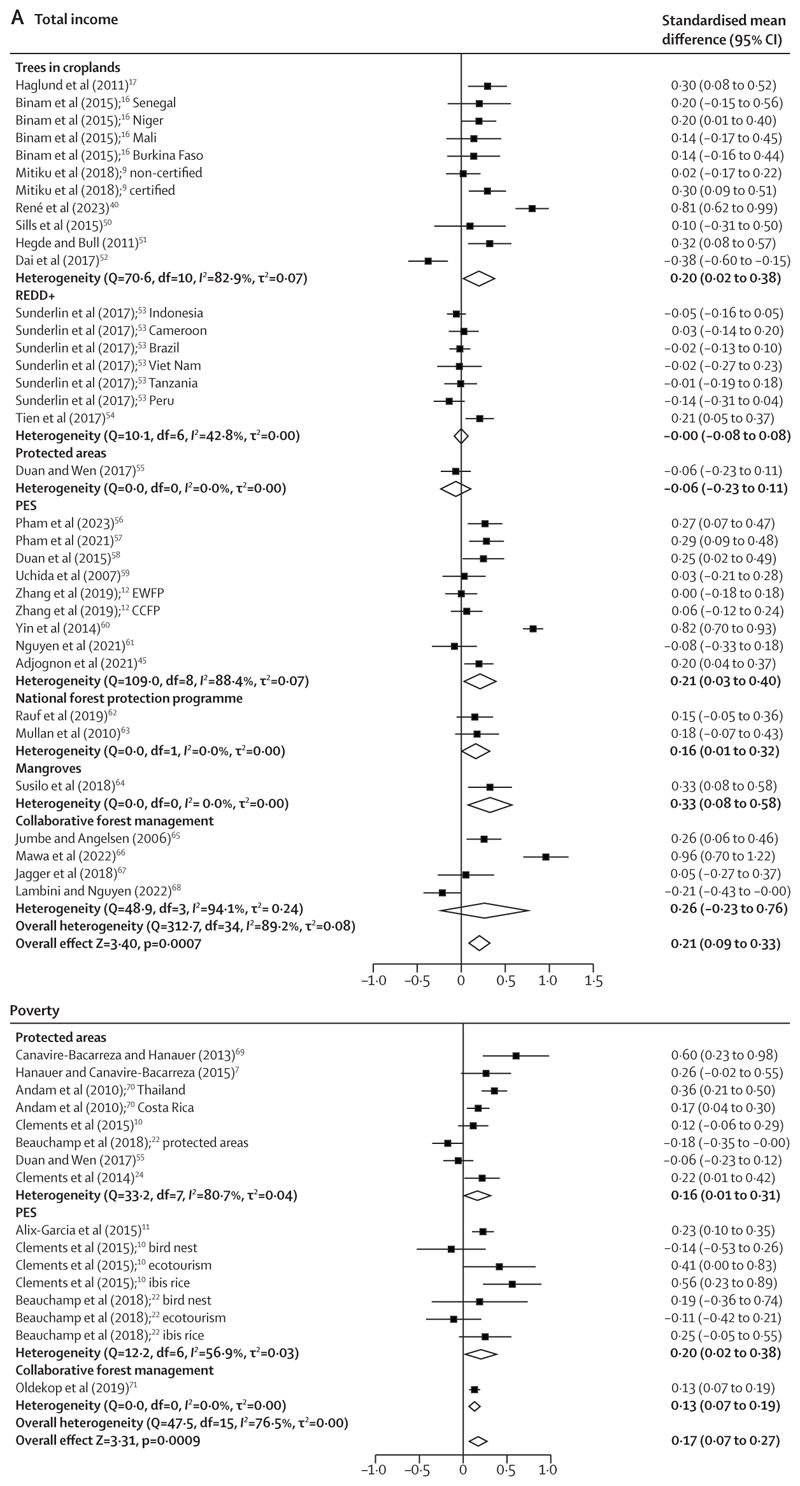

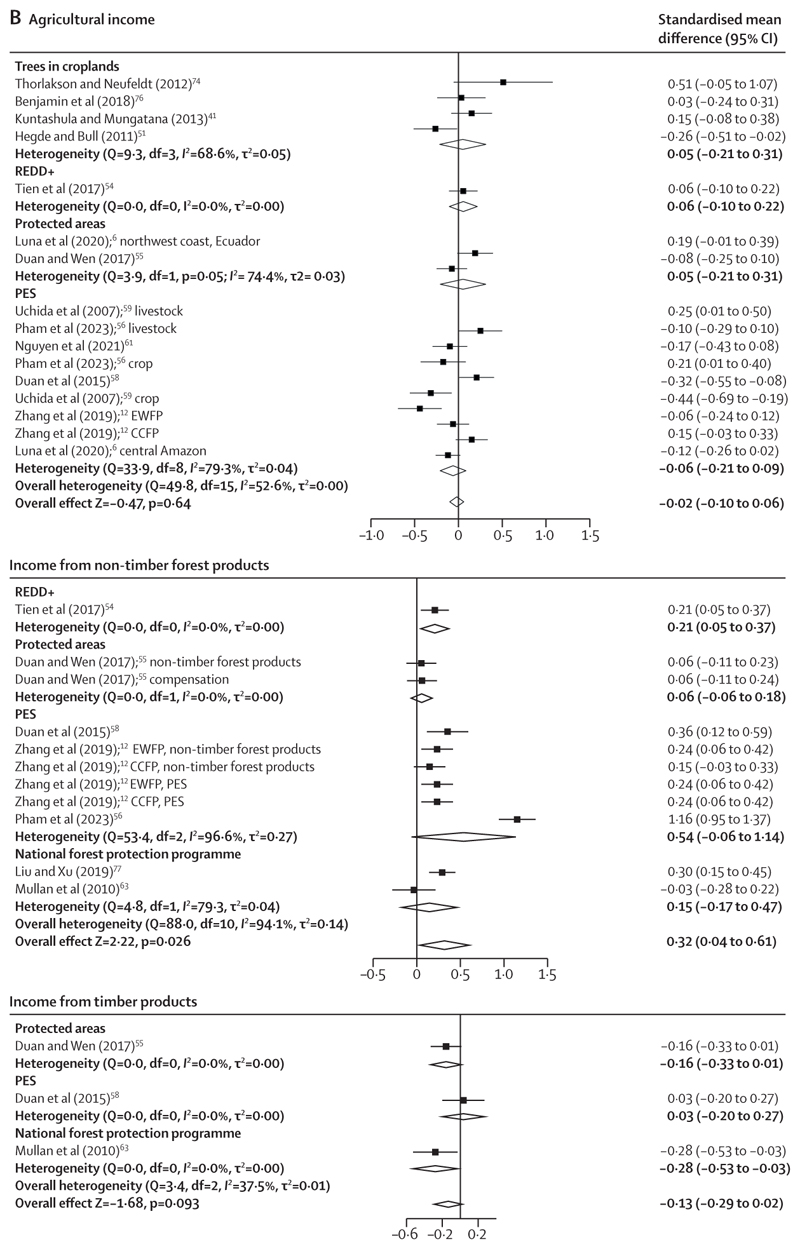
Forest plots of pooled and subgroup analysis effects of the impact of interventions on income and poverty reduction Impact of interventions on total income and poverty (A) and on agricultural income, income from non-timber forest products, and income from timber products (B). Positive effects indicate good outcomes. CCFP=conversion of croplands to forest programme. EWFP=ecological welfare forest programme. PES=payments for ecosystem services. REDD+=reducing emissions from deforestation and forest degradation in developing countries.

Income was the most studied of the outcomes, with 35 analyses across 25 studies evaluating the impact of interventions on total household income (figure 4A). These analyses showed positive, significant pooled effects (SMD 0·21 [95% CI 0·09 to 0·33], p=0·0007; *I*^2^=89·2% and τ^2^=0·08), with large variability by study and intervention type (figure 4A). The pooled effects on poverty, derived from 16 analyses from nine studies, were positive and significant (0·17 [0·07 to 0·27]; p=0·0009; *I*^2^=76·5% and τ^2^=0·00; figure 4A). Some studies reported on other financial dimensions that were thematically different from total income and poverty and were therefore not included in the meta-analysis on total income (figure 4A). These financial dimensions included outcomes related to household wealth (measured as total assets^11,47,72–74^ or physical or financial capital^75^) and employment.^62^ All effects on these outcomes were inconclusive other than positive and significant effects on household assets associated with a PES intervention in Mexico^73^ and an increase in employment prospects linked to a national forest protection programme in Pakistan.^62^

Three studies reported the effects on income from both timber production and non-timber forest products. The studies suggest that tree-based interventions could negatively affect income from timber production, although the pooled effects were from only three studies and were non-significant at 95% CI (−0·13 [−0·29 to 0·02]; p=0·093; *I*^2^=37·5% and τ^2^=0·01; figure 4B). It is probable that income from non-timber forest products could address, to some extent, the deficit generated by reduced timber-related revenue, as suggested by the effects pooled across seven studies (11 analyses) that reported on this outcome (0·32 [0·04 to 0·61]; p=0·026; *I*^2^=94·1%, τ^2^=0·14; figure 4B). Five studies^6,39,51,67,78^ reported the combined income from timber and non-timber forest products only and were therefore not included in the meta-analysis (figure 4B) that reported these effects separately. In these studies, the effect of REDD+,^78^ protected areas,^6^ and community forests^67^ on the combined income from timber and non-timber forest products was inconclusive, although one study on agroforestry^39^ found significant and positive effects.

No effect was observed on income from agricultural products (−0·02 [−0·10 to 0·06]; p=0·64; *I*2=52·6% and τ^2^=0·00), pooled from 16 analyses from 12 studies (figure 4B). A single study reported significance and positive effects on agricultural productivity (the difference between agricultural inputs and outputs) linked to a PES intervention in China.^79^

**Figure 5:**
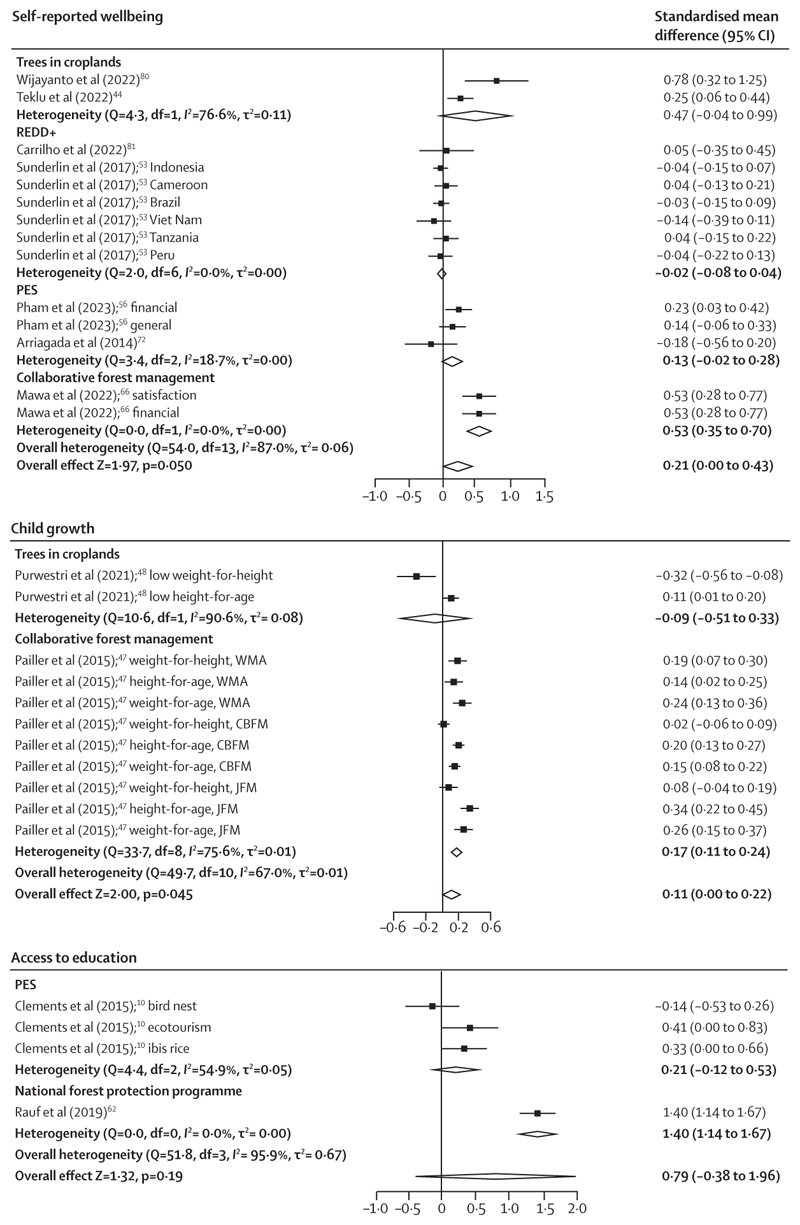
Forest plots of pooled and subgroup analysis effects of the impact of interventions on self-reported wellbeing, child growth, and access to education Positive effects indicate good outcomes. CBFM=community-based forest management. JFM=joint forest management. PES=payments for ecosystem services. REDD+=reducing emissions from deforestation and forest degradation in developing countries. WMA=wildlife management areas.

Positive effect sizes were observed for all child-growth outcomes except for low weight-for-height, which had a negative effect size (figure 5); however, the pooled effects for overall child-growth outcomes (from 11 analyses from two studies) were positive and significant (SMD 0·11 [95% CI 0·00 to 0·22], p=0·045; *I*^2^=67·0% and τ^2^=0·01).

The pooled effect sizes on self-reported wellbeing (from 14 analyses from seven studies) were also positive (0·21 [0·00 to 0·43]; p=0·049; *I*^2^=87·0% and τ^2^=0·06), with considerable variation by type of intervention (figure 5). The impact on access to education also varied by type of intervention: a study of a national forest protection programme found a significant and positive effect^62^ whereas a project that targeted biodiversity had no detectable effect^10^ (figure 5). The pooled effects on access to education were large but insignificant (0·79 [−0·38 to 1·96]; p=0·18; *I*^2^=95·9% and τ^2^=0·67), pooled from four analyses from two studies (figure 5). One study found a significant reduction in hospital admissions attributed to a PES intervention; this improvement in health probably contributed to increases in agricultural productivity.^79^

Egger’s test did not indicate funnel plot asymmetry and therefore did not suggest missing or unreported evidence (appendix pp 17–18), although some outcomes were reported in too few studies to enable funnel plot analyses using Egger’s regression.

## Discussion

Our meta-analysis, based on 54 studies, found that tree-based conservation solutions can have positive and significant impacts on outcomes related to agricultural yields, dietary diversity, income generation, poverty reduction, child growth, and self-reported wellbeing. The pooled effect sizes indicated no significant impact on food security; however, subgroup analyses found that interventions related to trees in croplands and PES increased food security, whereas those related to protected areas and collaborative forest management had no detectable effects. Potential income generated from non-timber forest products could help to ease any income shortfall related to a decline in timber production; the evidence suggested possible loss of income from timber production, although the pooled effects were non-significant. Impacts on income from agricultural products were inconclusive. These results highlight the potential for tree-based ecosystem solutions to provide benefits linked to human health and wellbeing while encouraging the sustainable use of forest resources, such as non-timber forest products.

The variation in effect sizes by intervention type, overall programme aims, and study location suggests that the outcomes of interventions are highly contextualised. In general, interventions that emphasised livelihood improvement and community development, such as trees in croplands and PES, had positive impacts on the outcomes examined whereas interventions that targeted biodiversity or the removal of greenhouse gases had unfavourable or no detectable effect on agricultural yields, food security, total household income, and wellbeing. Income was the most studied outcome, which is not surprising given the poverty-alleviation objectives of solutions implemented in LMIC settings; however, more studies are needed to measure losses in income from forestry and how households address these losses. Increased income could contribute to better health by facilitating access to goods and services. Increases in income and poverty are closely related and are probably linked to livelihood diversification,^12^ which can have a positive impact on health and wellbeing in rural LMIC settings.

Several limitations should be considered when interpreting our findings. First, the meta-analyses combine results from several interventions and practices (appendix p 2), and the large heterogeneity suggests differences in context and evaluation methods. Our subgroup analysis is a useful baseline for future work aimed at understanding contextual differences by type of practice. However, caution is advised when interpreting the evidence owing to some overlap in the interventions—eg, when PES programmes are co-located within protected areas or when these programmes used agroforestry techniques. Other moderating factors besides the type of intervention could explain the observed heterogeneity, including factors not examined in the meta-analysis such as the scale of the implementation, variation in effect by demographic or socioeconomic groups, or other factors not reported in the studies. The use of causal inference study designs to estimate impact also has methodological limitations, as indicated by our assessment of the risk of bias. We excluded studies that failed to appropriately define the intervention and those that did not match control and intervention sites; studies that matched sites without statistical matching were included and were marked as having a critical risk of bias. Additionally, many studies had cross-sectional designs, which do not account for unobservable time-variant characteristics and could incorrectly attribute positive effect sizes to an intervention.^53^ Several studies also used recall to measure outcomes before implementation, which was deemed less reliable on the basis of the length of recall; all studies were accordingly assessed for measurement error in the risk-of-bias assessment.

Translation of our findings into policy and practice requires some caution; nevertheless, these results have several implications for local and international policies. For example, our findings suggest that policy integration could be beneficial when developing National Biodiversity Strategies and Action Plans for LMICs, and these settings should increase efforts towards the cross-sectoral review of these plans and their implementation that occur every 2 years. The evidence highlights the need for policies to emphasise targeted technical and financial support for communities to diversify their income, especially communities that are transitioning from the production of timber to non-timber forest products. The findings also support the Nature’s Contribution to People framework, and specifically the good quality of life (human wellbeing) dimension of the Intergovernmental Science-Policy Platform on Biodiversity and Ecosystem Services framework.^82^ The capacity of trees to provide benefits depends on the health of the ecosystems in which they grow.^5^ Maximising health and wellbeing benefits therefore requires safeguarding the integrity of ecosystems (such as forests) and not merely planting single-standing trees. Interventions focused primarily on solutions to mitigate climate change or those that restrict access to natural resources continue to divide opinion.^8^ Despite well intentioned safeguards to protect livelihoods, our findings indicate that nature-based interventions in which the main objectives are climate mitigation (such as REDD_+_) or conservation of biodiversity (such as protected areas) still fall short of securing human health and wellbeing in terms of the outcomes we studied. We did not examine the implications of any of the interventions on equity as very few studies stratified effects by socioeconomic group, although some studies alluded to this: for example, REDD+ was found to widen inequality between participants and non-participants and by land ownership.^54^ In some instances, wealthier households benefited more than poorer ones from collaborative forest management and PES programmes.^47,59^

Our review offers compelling evidence for the potential of tree-based solutions to address health outcomes. However, considerable gaps remain in generating quantitative measures for health outcomes beyond those empirically studied to date. For example, qualitative assessments show that higher upstream tree cover is associated with a lower probability of diarrhoeal diseases downstream^83^ and that farmer-led natural regeneration reportedly has positive impacts on heat adaptation, air and water quality, mental health, and waterborne diseases;^84^ however, as yet, quantitative data are not available for analysis. Solutions that achieve impact through reduction of inequality also warrant future research. Few studies examine health and wellbeing alongside ecological outcomes to improve our understanding of positive interactions between humans and nature and what conditions and context lead to them. Socioecological frameworks used alongside rigorous impact and process evaluations are crucial tools in decoupling complex interactions: attributing cause and effect, providing insight on mechanisms through which effects take place, and identifying the populations that benefit the most and the least. However, the application of these methods remains extremely scarce, in part because of the methodological challenges we have discussed.

Unlocking the full potential of nature-based solutions will require integrating health and wellbeing objectives alongside ecosystem restoration goals. In practice, such integration will require consideration of population needs when making decisions on land use and conservation; likewise, decisions on population health will need oversight of environmental exposure pathways that have been shown to improve wellbeing or to generate or exacerbate illness. The positive effect sizes shown in this Review are an important step towards generating evidence that integrates health and ecosystem services. For example, the causal relationship between forest conservation and nutritional outcomes strengthens the case for integrating forest management and nutrition interventions.^85^ Robust and meaningful integration will require the study of outcomes that closely align with health objectives. General wellbeing measures, although informative, are too broad and complex^86^ and could lack direct relevance for health studies. Instead, evaluating specific outcomes, such as impacts on illness or mortality, would provide more practical insights for health applications. Generating such evidence will need considerable input from health professionals beyond current levels: of the 54 studies included in the meta-analysis, only one was led by an author with a health background, suggesting that we are a long way from using cross-disciplinary investigations to comprehensively characterise the links between ecosystem restoration and human health. Because achieving health outcomes will coincide with meeting several of the UN’s Sustainable Development Goals, demonstrating health benefits could be a powerful lever for mobilising public and political support for conservation.

## Supplementary Material

Supplementary appendix

## Data Availability

All meta-analyses reported in this study—including data extracted from included studies, data used for analyses, and analytical code—are available on request to the corresponding author.
