# Supplementary material for: Impact of tree-based interventions in addressing health and wellbeing outcomes in rural low-income and middle-income settings: a systematic review and meta-analysis
Source: Lancet Planet Health. Author manuscript; Available in PMC 2025 Dec 6. (PMC7618441; doi:10.1016/S2542-5196(25)00003-8)
Supplement: Supplementary appendix [file EMS211167-supplement-Supplementary_appendix.pdf]

THE LANCET
Planetary Health

Supplementary appendix

This appendix formed part of the original submission and has been peer reviewed. We post it as supplied by the authors.

Supplement to: Murage P, Anton B, Chiwanga F, et al. Impact of tree-based interventions in addressing health and wellbeing outcomes in rural low-income and middle-income settings: a systematic review and meta-analysis. *Lancet Planet Health* 2025; **9:** e157–68.

Supplementary Information

Supplementary to: Impact of tree-based interventions in addressing health and wellbeing outcomes in rural low-and-middle-income settings: a systematic review and meta-analysis

Authors: Peninah Murage[1], Blanca Anton[1], Faraja Chiwanga[2], Roberto Picetti[1], Tabby Njunge[3], Syreen Hassan[1], Sarah Whitmee[1], Jane Falconer[1], Hugh Sharma Waddington[1] and Rosemary Green[1]

Affiliations:

1. London School of Hygiene and Tropical Medicine, UK

2. Muhimbili National Hospital University of Health and Allied Sciences and Leadership Formation, Environmental Conservation and Action for Development (LEAD) Foundation, Tanzania

3. Conservation International, Kenya

* Corresponding author: Dr Peninah Murage, London School of Hygiene & Tropical Medicine, Keppel St, London WC1E 7HT, UK: Peninah.Murage@lshtm.ac.uk

Table of Contents

Appendix A: Theory of Change Linking Ecosystem Restoration to Health and Wellbeing Outcomes 1

Appendix B: Implemented tree-based interventions – broad categories and discussion on overlaps 2

Appendix C: Search Strategy for Web of Science 3

Appendix D: EPPI-Reviewer Priority Screening 7

Figure D2 – EPPI-Reviewer example of classifier bands 7

Appendix E: PRISMA Checklist 8

Appendix F: Approach to risk of bias assessment 10

Figure F1: Overall risk of bias 10

Figure F2: Study level risk of bias 11

Appendix G: List of included studies summarised by type of intervention, implementation country, study design and risk of bias assessment summary score 12

Appendix H: Models without adjustment for effect dependence 16

Appendix I: Funnel plots and Egger's regression test for funnel plot asymmetry 17

Appendix A: Theory of Change Linking Ecosystem Restoration to Health and Wellbeing Outcomes

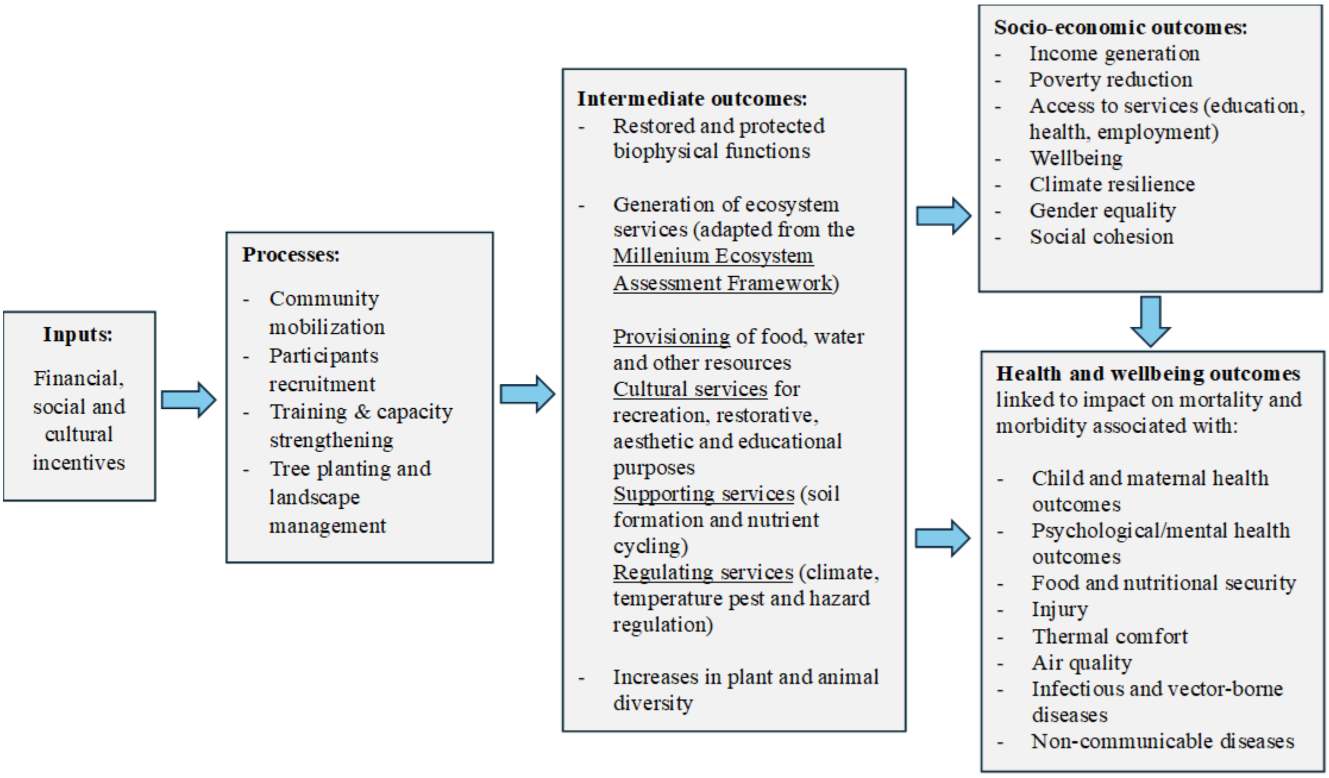

Appendix B: Implemented tree-based interventions – broad categories and discussion on overlaps

Collaborative Forest Management: This category describes the management of forests with or by local communities with varying elements of collective activity and land tenure[37]. The following interventions were included; community forest management initiatives, co-management programs which promote local participation in forest management and sustainable use of forest resources, and smallholder forestry where land is individually owned but managed via collective action.

Payment for Ecosystem Services or Payment for Environmental Services (PES): Payment for Ecosystem service is an agreement that seeks to create market-driven incentives either cash or in-kind in environmental management[19]. In this review, we defined PES interventions as those that involve a series of payments to land or other natural resource owners in return for a guaranteed flow of ecosystem services over and above what would otherwise be provided in the absence of payment[20].

Protected Areas (PA): We applied the IUCN definition of Protected Areas which defines these as geographical areas dedicated to the protection and maintenance of biological diversity, and natural and associated cultural resources, and managed through legal or other effective means[18]. There were some overlaps in the Protected Areas and PES interventions in some of the included studies[10,22] where PES programs were co-located within Protected Areas.

Trees in croplands: This broadly refers to trees on cropland and includes the management of appropriate species on farmland including woody perennials. We included agroforestry systems such as agrisilvicultural practices (intercropping crops and trees) silvipastoral where forestry and pastureland are combined, and natural regeneration restoration techniques such as 'Farmer Managed Natural Regeneration (FMNR) that involve the regrowth and management of existing (usually indigenous) trees and shrubs from felled tree stumps, sprouting root systems, or seeds[15-17]. We found some overlaps in agroforestry interventions and PES where some landowners were incentivized to integrate trees in their landscapes.

REDD+ *(Reducing Emissions from Deforestation and forest Degradation, plus the sustainable management of forests, and the conservation and enhancement of forest carbon stocks):* This is a climate change mitigation solution developed by parties to the United Nations Framework Convention on Climate (UNFCCC)[21]. It recognizes the important role of forests in mitigating climate change through the removal of carbon from the atmosphere and storage of biomass and soils. It also aims to halt the clearing and degradation of forests which are a source of greenhouse gas emissions when stored carbon is released. There are some overlaps with PES where landowners earn revenue through forest carbon credits through reduction of emissions and carbon removal and storage.

National Forest Protection Program: The included interventions took a strategic country-level approach towards sustainable management, conservation, restoration, and utilization of forests and associated resources for socioeconomic growth and climate resilience. Activities include the development of supporting policies, planning, implementation, monitoring, and evaluation of identified sustainable forest management activities and actions at national and subnational levels[38].

Appendix C: Search Strategy for Web of Science

15
#14 Editions: WOS.ESCI,WOS.SCI,WOS.SSCI Timespan: 2000-01-01 to 2023-12-31

14
#1 and #2 and #5 and #12 and #13

13
TI=("impact" OR "outcome" OR "effect*" OR "intervention$" OR "evaluation$" OR "assessment" OR "effective*" OR "ineffective*" OR "cost-benefit*" OR "efficacy" OR "observational stud*" OR "propensity score match*" OR "regression" OR "difference in difference" OR "matching" OR "instrumental variable*" OR "comparison" OR "counterfactual" OR "counter-factual" OR "quasi-experimental" OR "quasiexperimental" OR (("quantitative" or "experiment*") near/2 ("design" or "study" or "studies" or "analys*")) OR "cross-sectional" or "prevalence" OR "trial" OR "RCT" OR "model*" or "case-study" or "case-studies") OR AB=("impact" OR "outcome" OR "effect*" OR "intervention$" OR "evaluation$" OR "assessment" OR "effective*" OR "ineffective*" OR "cost-benefit*" OR "efficacy" OR "observational stud*" OR "propensity score match*" OR "regression" OR "difference in difference" OR "matching" OR "instrumental variable*" OR "comparison" OR "counterfactual" OR "counter-factual" OR "quasi-experimental" OR "quasiexperimental" OR (("quantitative" or "experiment*") near/2 ("design" or "study" or "studies" or "analys*")) OR "cross-sectional" or "prevalence" OR "trial" OR "RCT" OR "model*" or "case-study" or "case-studies")

12
#6 or #7 or #8 or #9 or #10 or #11

11
TI=("impact" OR "outcome" OR "effect*" OR "intervention$" OR "evaluation$" OR "assessment" OR "effective*" OR "ineffective*" OR "cost-benefit*" OR "efficacy" OR "observational stud*" OR "propensity score match*" OR "regression" OR "difference in difference" OR "matching" OR "instrumental variable*" OR "comparison" OR "counterfactual" OR "counter-factual" OR "quasi-experimental" OR "quasiexperimental" OR (("quantitative" or "experiment*") near/2 ("design" or "study" or "studies" or "analys*")) OR "cross-sectional" or "prevalence" OR "trial" OR "RCT" OR "model*" or "case-study" or "case-studies") OR AB=("impact" OR "outcome" OR "effect*" OR "intervention$" OR "evaluation$" OR "assessment" OR "effective*" OR "ineffective*" OR "cost-benefit*" OR "efficacy" OR "observational stud*" OR "propensity score match*" OR "regression" OR "difference in difference" OR "matching" OR "instrumental variable*" OR "comparison" OR "counterfactual" OR "counter-factual" OR "quasi-experimental" OR "quasiexperimental" OR (("quantitative" or "experiment*") near/2 ("design" or "study" or "studies" or "analys*")) OR "cross-sectional" or "prevalence" OR "trial" OR "RCT" OR "model*" or "case-study" or "case-studies")

10
TI=("africa south of the sahara" or "sub-saharan africa" or "central africa" or "east* africa" or "southern africa" or "west* africa" or "sahel" OR "benin" or "dahomey" or "burkina faso" or "burkina fasso" or "upper volta" or "burundi" or "central african republic" or "ubangi-shari" or "chad" or "comoros" or "comoro islands" or "mayotte" or "iles comores" or ("democratic republic" near/2 "congo") or "belgian congo" or "zaire" or "eritrea" or "ethiopia" or "gambia" or ("guinea" not ("new guinea" or "guinea pig*" or "guinea fowl")) or "guinea-bissau" or "portuguese guinea" or "liberia" or "madagascar" or "malagasy republic" or "malawi" or "nyasaland" or "mali" or "mozambique" or "mocambique" or "portuguese east africa" or ("niger" not (Aspergillus or Peptococcus or Schizothorax or Cruciferae or Gobius or Lasius or Agelastes or Melanosuchus or radish or Parastromateus or Orius or Apergillus or Parastromateus or Stomoxys)) or "rwanda" or "ruanda" or "senegal" or "sierra leone" or "somalia" or "south sudan" or "tanzania" or "tanganyika" or "zanzibar" or "togo" or "togolese republic" or "uganda" or "zimbabwe" or "rhodesia" or "angola" or "cameroon" or "cape verde" or "cabo verde" or ("congo" not (("democratic republic" near/3 congo) or "congo red" or "crimean congo")) or "cote d ivoire" or "cote divoire" or "ivory coast" or "ghana" or "gold coast" or "kenya" or "lesotho" or "basutoland" or "mauritania" or "nigeria" or ("sao tome" near/2 "principe") or ("sudan" not "south sudan") or "eswatini" or "swaziland" or "zambia" or "northern rhodesia" or "botswana" or "bechuanaland" or "kalahari" or "equatorial guinea" or "spanish guinea" or "gabon" or "gabonese republic" or "mauritius" or "agalega islands" or "namibia" or "south africa")

9

AB=("africa south of the sahara" or "sub-saharan africa" or "central africa" or "east* africa" or "southern africa" or "west* africa" or "sahel" OR "benin" or "dahomey" or "burkina faso" or "burkina fasso" or "upper volta" or "burundi" or "central african republic" or "ubangi-shari" or "chad" or "comoros" or "comoro islands" or "mayotte" or "iles comores" or ("democratic republic" near/2 "congo") or "belgian congo" or "zaire" or "eritrea" or "ethiopia" or "gambia" or ("guinea" not ("new guinea" or "guinea pig*" or "guinea fowl")) or "guinea-bissau" or "portuguese guinea" or "liberia" or "madagascar" or "malagasy republic" or "malawi" or "nyasaland" or "mali" or "mozambique" or "mocambique" or "portuguese east africa" or ("niger" not (Aspergillus or Peptococcus or Schizothorax or Cruciferae or Gobius or Lasius or Agelastes or Melanosuchus or radish or Parastromateus or Orius or Apergillus or Parastromateus or Stomoxys)) or "rwanda" or "ruanda" or "senegal" or "sierra leone" or "somalia" or "south sudan" or "tanzania" or "tanganyika" or "zanzibar" or "togo" or "togolese republic" or "uganda" or "zimbabwe" or "rhodesia" or "angola" or "cameroon" or "cape verde" or "cabo verde" or ("congo" not (("democratic republic" near/3 congo) or "congo red" or "crimean congo")) or "cote d ivoire" or "cote divoire" or "ivory coast" or "ghana" or "gold coast" or "kenya" or "lesotho" or "basutoland" or "mauritania" or "nigeria" or ("sao tome" near/2 "principe") or ("sudan" not "south sudan") or "eswatini" or "swaziland" or "zambia" or "northern rhodesia" or "botswana" or "bechuanaland" or "kalahari" or "equatorial guinea" or "spanish guinea" or "gabon" or "gabonese republic" or "mauritius" or "agalega islands" or "namibia" or "south africa")

8
TI=("haiti" or "bolivia" or "el salvador" or "guatemala" or "honduras" or "nicaragua" or "argentina" or "belize" or "brazil" or "colombia" or "costa rica" or "cuba" or "dominica" or "dominican republic" or "ecuador" or "grenada" or "guyana" or "jamaica" or "mexico" or "panama" or "paraguay" or "peru" or "saint lucia" or "st lucia" or "grenadines" or "suriname" or "venezuela") or AB= ("haiti" or "bolivia" or "el salvador" or "guatemala" or "honduras" or "nicaragua" or "argentina" or "belize" or "brazil" or "colombia" or "costa rica" or "cuba" or "dominica" or "dominican republic" or "ecuador" or "grenada" or "guyana" or "jamaica" or "mexico" or "panama" or "paraguay" or "peru" or "saint lucia" or "st lucia" or "grenadines" or "suriname" or "venezuela")

7
TI=("armenia" or "kosovo" or ("georgia" near/2 "republic") or "kosovo" or "kyrgyzstan" or "kyrgyz republic" or "kirghizia" or "kirghiz" or "moldova" or "tajikistan" or "ukraine" or "uzbekistan" or "albania" or "azerbaijan" or "belarus" or "byelarus" or "belorussia" or "bosnia" or "herzegovina" or "bulgaria" or "kazakhstan" or "kazakh" or "macedonia" or "montenegro" or "romania" or "russia" or "ussr" or "russian federation" or "union of soviet socialist republics" or "soviet union" or "serbia" or "turkey" or "turkmenistan" or "yugoslavia" or "djibouti" or "french somaliland" or "egypt" or "jordan" or "morocco" or "syria" or "syrian arab republic" or "tunisia" or "gaza" or "yemen" or "algeria" or "iran" or "iraq" or "lebanon" or "libya") or AB=("armenia" or "kosovo" or ("georgia" near/2 "republic") or "kosovo" or "kyrgyzstan" or "kyrgyz republic" or "kirghizia" or "kirghiz" or "moldova" or "tajikistan" or "ukraine" or "uzbekistan" or "albania" or "azerbaijan" or "belarus" or "byelarus" or "belorussia" or "bosnia" or "herzegovina" or "bulgaria" or "kazakhstan" or "kazakh" or "macedonia" or "montenegro" or "romania" or "russia" or "ussr" or "russian federation" or "union of soviet socialist republics" or "soviet union" or "serbia" or "turkey" or "turkmenistan" or "yugoslavia" or "djibouti" or "french somaliland" or "egypt" or "jordan" or "morocco" or "syria" or "syrian arab republic" or "tunisia" or "gaza" or "yemen" or "algeria" or "iran" or "iraq" or "lebanon" or "libya")

6
TI=("north korea" or ("democratic people* republic" near/2 "korea") or "cambodia" or "indonesia" or "kiribati" or "laos" or ("lao" near/1 "democratic republic") or "micronesia" or "mongolia" or "myanmar" or "burma" or "papua new guinea" or "philippines" or "solomon islands" or "timor-leste" or "vanuatu" or "viet nam" or "vietnam" or "american samoa" or "china" or "fiji" or "malaysia" or "marshall islands" or "nauru" or "independent state of samoa" or "western samoa" or "navigator islands" or "samoan islands" or "thailand" or "tonga" or "tuvalu" or "melanesia" or "polynesia" or "afghanistan" or "nepal" or "bangladesh" or "bhutan" or "india" or "pakistan" or "sri lanka" or "ceylon" or "maldives") or AB=("north korea" or ("democratic people* republic" near/2 "korea") or "cambodia" or "indonesia" or "kiribati" or "laos" or ("lao" near/1 "democratic republic") or "micronesia" or "mongolia" or "myanmar" or "burma" or "papua new guinea" or "philippines" or "solomon islands" or "timor-leste" or "vanuatu" or "viet nam" or "vietnam" or "american samoa" or "china" or "fiji" or "malaysia" or "marshall islands" or "nauru" or "independent state of samoa" or "western samoa" or "navigator islands" or "samoan islands" or "thailand" or "tonga" or "tuvalu" or "melanesia" or "polynesia" or "afghanistan" or "nepal" or "bangladesh" or "bhutan" or "india" or "pakistan" or "sri lanka" or "ceylon" or "maldives")

5

TI=((("developing" or "less* developed" or "under developed" or "underdeveloped" or "middle income" or "low* income" or "underserved" or "under-served" or "deprived" or poor*) near/2 ("economy" or "economies" or countr* or nation? or population? or "world")) or (low* near/2 ("gdp" or "gnp" or "gross domestic" or "gross national")) or "lmic" or "lmics" or "third world" or "lami countr*" or "transitional countr*" or "global south" or "majority world") or AB=((("developing" or "less* developed" or "under developed" or "underdeveloped" or "middle income" or "low* income" or "underserved" or "under-served" or "deprived" or poor*) near/2 ("economy" or "economies" or countr* or nation? or population? or "world")) or (low* near/2 ("gdp" or "gnp" or "gross domestic" or "gross national")) or "lmic" or "lmics" or "third world" or "lami countr*" or "transitional countr*" or "global south" or "majority world")

4
TI=("socio-economic" or "environmental-economic" or "social-ecological" or "economic*" or "income*" or "livelihood*" or "wage*" or "poor" or "poverty" or "low-income" or "wealth" or "standard of living" or "subsistence" or "employment" or "unemployment" or "employed" or "unemployed" or "cash" or "pay*" or "monetary" or "money" or "allowance" or "voucher*" or "enterpris*" OR "micro-enterpris*" or ("crop" near/1 ("sale" or "sold" or "produc*")) or "yield*" OR "harvest*" or "agricultural develop*" OR "fuel*" OR "fuelwood*" OR "timber" OR "disaster risk reduction" OR ("disaster" NEAR/1 ("mitigat*" OR "reduc*" OR "protect*")) OR "adapt*" OR "resilie*" OR "alleviat*" OR "diversif*" OR "vulnerab*" OR "cope" OR "coping" OR "coped" OR "copes" OR (("food" or "water") NEAR/1 ("security" or "insecurity")) OR "equity" OR "inequal*" OR (("gender*" OR "female") NEAR/2 ("empower*" OR "equality" OR "violence")) OR ("domestic" NEAR/1 ("chores" OR "task*" OR "work*" OR "labo$r" OR "violence")) OR "conflict" OR "migrat*" OR "internal displace*" OR "school*" OR "educat*" OR "network*" OR ("capital" NEAR/1 ("social" OR "human" OR "natural")) or diversif*) OR AB=("socio-economic" or "environmental-economic" or "social-ecological" or "economic*" or "income*" or "livelihood*" or "wage*" or "poor" or "poverty" or "low-income" or "wealth" or "standard of living" or "subsistence" or "employment" or "unemployment" or "employed" or "unemployed" or "cash" or "pay*" or "monetary" or "money" or "allowance" or "voucher*" or "enterpris*" OR "micro-enterpris*" or ("crop" near/1 ("sale" or "sold" or "produc*")) or "yield*" OR "harvest*" or "agricultural develop*" OR "fuel*" OR "fuelwood*" OR "timber" OR "disaster risk reduction" OR ("disaster" NEAR/1 ("mitigat*" OR "reduc*" OR "protect*")) OR "adapt*" OR "resilie*" OR "alleviat*" OR "diversif*" OR "vulnerab*" OR "cope" OR "coping" OR "coped" OR "copes" OR (("food" or "water") NEAR/1 ("security" or "insecurity")) OR "equity" OR "inequal*" OR (("gender*" OR "female") NEAR/2 ("empower*" OR "equality" OR "violence")) OR ("domestic" NEAR/1 ("chores" OR "task*" OR "work*" OR "labo$r" OR "violence")) OR "conflict" OR "migrat*" OR "internal displace*" OR "school*" OR "educat*" OR "network*" OR ("capital" NEAR/1 ("social" OR "human" OR "natural")) or diversif*)

3
TI= ("health*" or "unhealth*" or "welfare" or "well-being" or "wellbeing" or "wellness" or ("life" near/2 "satisf*") or "diet" or "dietary" or "nutrition*" or "hunger") or AB=("health*" or "unhealth*" or "welfare" or "well-being" or "wellbeing" or "wellness" or ("life" near/2 "satisf*") or "diet" or "dietary" or "nutrition*" or "hunger")

2
TI = (("tree*" NOT ((decision or regression or statistic*) near/1 tree*)) OR "wood" or "woody" OR ("forest*" NOT (forest near/1 (plot or random or isolation or "random survival"))) OR "canop*" OR "understory" OR "shrub*" OR "mangrove*" OR "parkland*" OR "agroforest*" OR "agro-forest*" OR "agro-pastor*" OR "agropastor*" OR "silvopastor*" OR "silvipastor*" OR "agrisilviculture*" OR "aqua-silvo*" OR "exclosure" OR "boundary planting" OR "hedgerow*" OR "improved fallows" OR "shadow system*" OR "living fence*" OR "entomoforest*" OR "homegarden*" OR "home-garden*" OR "shade-species" OR "shade-grown" OR "farmer managed natural restoration" OR "FMNR" OR "REDD" OR "REDD+" OR "afforest*" OR "reforest*" OR "deforest*") OR AB = (("tree*" NOT ((decision or regression or statistic*) near/1 tree*)) OR "wood" OR "woody" OR ("forest*" NOT (forest near/1 (plot or random or isolation or "random survival"))) OR "canop*" OR "understory" OR "shrub*" OR "mangrove*" OR "parkland*" OR "agroforest*" OR "agro-forest*" OR "agro-pastor*" OR "agropastor*" OR "silvopastor*" OR "silvipastor*" OR "agrisilviculture*" OR "aqua-silvo*" OR "exclosure" OR "boundary planting" OR "hedgerow*" OR "improved fallows" OR "shadow system*" OR "living fence*" OR "entomoforest*" OR "homegarden*" OR "home-garden*" OR "shade-species" OR "shade-grown" OR "farmer managed natural restoration" OR "FMNR" OR "REDD" OR "REDD+" OR "afforest*" OR "reforest*" OR "deforest*")

1

TI = ("household*" OR "smallhold*" OR "small-hold*" OR "farm" OR "farms" OR "farmer*" OR "village*" OR "rural" OR "non-urban" OR coast* OR ("communit*" not (communit* near/1 (plant* or vegetat* or ecolog* or ecosyst* or biodivers* or wild))) OR "neighbo$rhood") OR AB = ("household*" OR "smallhold*" OR "small-hold*" OR "farm" OR "farms" OR "farmer*" OR "village*" OR "rural" OR "non-urban" OR ("communit*" not (communit* near/1 (plant* or vegetat* or ecolog* or ecosyst* or biodivers* or wild))) OR "neighbo$rhood")

Appendix D: EPPI-Reviewer Priority Screening

Priority Screening tool works by learning the characteristics of included and excluded studies to predict whether a given record is more likely to be relevant or irrelevant (Figure D1). The blue dots indicate 'irrelevant' records and the red dots indicate 'relevant records'. In traditional screening, the relevant records are usually distributed at random (top panel), but priority screening works by drawing out the relevant records towards the beginning of the screening process and 'pushing' the irrelevant ones towards the end (bottom panel).

Figure D1 Priority Screening tool

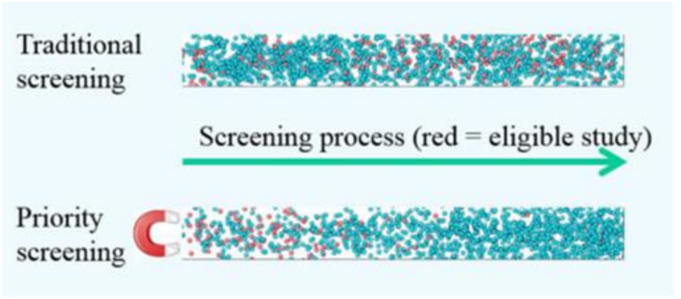

(Figure reproduced with permission by EPPI-Reviewer Team. Source: https://eppi.ioe.ac.uk/CMS/Portals/35/machine_learning_in_eppi-reviewer_v_7_web_version.pdf , originally at Gough et al 2017[32])

Each record is given a probability score and banded into deciles based on the likelihood to be relevant. Figure D2 gives an example of classifier bands that range from 0-9% (very likely to be relevant) and 90-99% (very likely to be irrelevant). Every screening iteration generated similar bands and screening was conducted on the lowest bands. This served two purposes, firstly we were able to identify studies that met the inclusion criteria, and secondly, it was a way of training the software to identify similar studies. The process was repeated several times until the lower bands no longer had relevant studies – this marked the point of saturation. In the event some records were missed, we concluded the exercise with a rapid title screening of all the excluded records.

Figure D2 – EPPI-Reviewer example of classifier bands

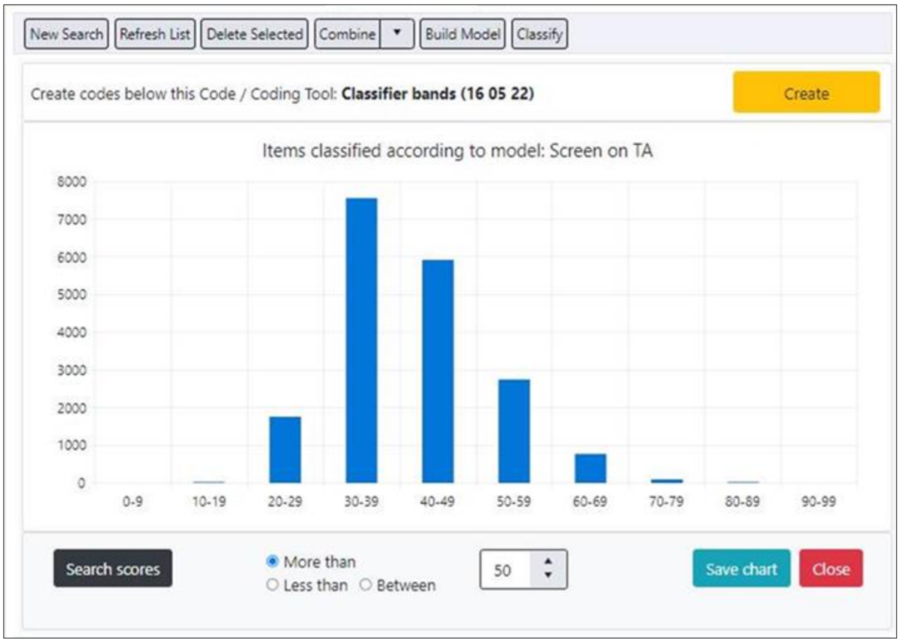

Appendix E: PRISMA Checklist

Section and Topic	Item #	Checklist item	Page where located
**TITLE**			
Title	1	Identify the report as a systematic review.	1 - 2
**ABSTRACT**			
Abstract	2	See the PRISMA 2020 for Abstracts checklist.	5
**INTRODUCTION**			
Rationale	3	Describe the rationale for the review in the context of existing knowledge.	2-3
Objectives	4	Provide an explicit statement of the objective(s) or question(s) the review addresses.	4
**METHODS**			
Eligibility criteria	5	Specify the inclusion and exclusion criteria for the review and how studies were grouped for the syntheses.	4
Information sources	6	Specify all databases, registers, websites, organisations, reference lists and other sources searched or consulted to identify studies. Specify the date when each source was last searched or consulted.	4
Search strategy	7	Present the full search strategies for all databases, registers and websites, including any filters and limits used.	4, Appendix C and reference # 24
Selection process	8	Specify the methods used to decide whether a study met the inclusion criteria of the review, including how many reviewers screened each record and each report retrieved, whether they worked independently, and if applicable, details of automation tools used in the process.	5
Data collection process	9	Specify the methods used to collect data from reports, including how many reviewers collected data from each report, whether they worked independently, any processes for obtaining or confirming data from study investigators, and if applicable, details of automation tools used in the process.	5
Data items	10a	List and define all outcomes for which data were sought. Specify whether all results that were compatible with each outcome domain in each study were sought (e.g. for all measures, time points, analyses), and if not, the methods used to decide which results to collect.	6
	10b	List and define all other variables for which data were sought (e.g. participant and intervention characteristics, funding sources). Describe any assumptions made about any missing or unclear information.	5-6
Study risk of bias assessment	11	Specify the methods used to assess risk of bias in the included studies, including details of the tool(s) used, how many reviewers assessed each study and whether they worked independently, and if applicable, details of automation tools used in the process.	5 & Appendix F
Effect measures	12	Specify for each outcome the effect measure(s) (e.g. risk ratio, mean difference) used in the synthesis or presentation of results.	5-6
Synthesis methods	13a	Describe the processes used to decide which studies were eligible for each synthesis (e.g. tabulating the study intervention characteristics and comparing against the planned groups for each synthesis (item #5)).	6
	13b	Describe any methods required to prepare the data for presentation or synthesis, such as handling of missing summary statistics, or data conversions.	5-6
	13c	Describe any methods used to tabulate or visually display results of individual studies and syntheses.	6
	13d	Describe any methods used to synthesize results and provide a rationale for the choice(s). If meta-analysis was performed, describe the model(s), method(s) to identify the presence and extent of statistical heterogeneity, and software package(s) used.	6
	13e	Describe any methods used to explore possible causes of heterogeneity among study results (e.g. subgroup analysis, meta-regression).	6
	13f	Describe any sensitivity analyses conducted to assess robustness of the synthesized results.	6
Reporting bias assessment	14	Describe any methods used to assess risk of bias due to missing results in a synthesis (arising from reporting biases).	6

Section and Topic	Item #	Checklist item	Page where located
Certainty assessment	15	Describe any methods used to assess certainty (or confidence) in the body of evidence for an outcome.	6
**RESULTS**			
Study selection	16a	Describe the results of the search and selection process, from the number of records identified in the search to the number of studies included in the review, ideally using a flow diagram.	7, Figure 1
	16b	Cite studies that might appear to meet the inclusion criteria, but which were excluded, and explain why they were excluded.	7, Figure 1
Study characteristics	17	Cite each included study and present its characteristics.	Appendix G
Risk of bias in studies	18	Present assessments of risk of bias for each included study.	Appendix G
Results of individual studies	19	For all outcomes, present, for each study: (a) summary statistics for each group (where appropriate) and (b) an effect estimate and its precision (e.g. confidence/credible interval), ideally using structured tables or plots.	Figures 3, 4 & 5
Results of syntheses	20a	For each synthesis, briefly summarise the characteristics and risk of bias among contributing studies.	8, Appendix G
	20b	Present results of all statistical syntheses conducted. If meta-analysis was done, present for each the summary estimate and its precision (e.g. confidence/credible interval) and measures of statistical heterogeneity. If comparing groups, describe the direction of the effect.	Figures 3, 4 & 5
	20c	Present results of all investigations of possible causes of heterogeneity among study results.	9-10
	20d	Present results of all sensitivity analyses conducted to assess the robustness of the synthesized results.	9-10
Reporting biases	21	Present assessments of risk of bias due to missing results (arising from reporting biases) for each synthesis assessed.	10, Appendix I
Certainty of evidence	22	Present assessments of certainty (or confidence) in the body of evidence for each outcome assessed.	Appendix F, Figure F1
**DISCUSSION**			
Discussion	23a	Provide a general interpretation of the results in the context of other evidence.	9
	23b	Discuss any limitations of the evidence included in the review.	9
	23c	Discuss any limitations of the review processes used.	9
	23d	Discuss implications of the results for practice, policy, and future research.	9-10
**OTHER INFORMATION**			
Registration and protocol	24a	Provide registration information for the review, including register name and registration number, or state that the review was not registered.	5
	24b	Indicate where the review protocol can be accessed, or state that a protocol was not prepared.	5
	24c	Describe and explain any amendments to information provided at registration or in the protocol.	5
Support	25	Describe sources of financial or non-financial support for the review, and the role of the funders or sponsors in the review.	6
Competing interests	26	Declare any competing interests of review authors.	
Availability of data, code and other materials	27	Report which of the following are publicly available and where they can be found: template data collection forms; data extracted from included studies; data used for all analyses; analytic code; any other materials used in the review.	10

Appendix F: Approach to risk of bias assessment

We used an existing critical appraisal tool[34] for randomised and non-randomised studies of effects that uses signalling questions assess to the risk of bias against the following seven domains.

1. **Confounding bias** examined whether the allocation or identification mechanism can address confounding, for example by adequately matching treated and control sites.
2. **Selection bias** into the study examined whether any differential selection into the study was adequately resolved, for example through random sampling.
3. **Attrition bias** (selection bias out of the study) examined whether differential selection out of the study was adequately resolved.
4. **Motivation bias** examined whether the process of observation had a low risk of 'Hawthorne effect' for example by blinding participants and outcome assessors. Cross-sectional studies were given a low risk for this bias.
5. **Performance bias** examined whether the study was adequately protected against spillovers, contamination, or crossovers.
6. **Measurement error** examined whether the study was free from biases in measurement, for example by ensuring participation in the intervention is observed, or the intervention is clearly and consistently defined and misreporting by participants or enumerators is unlikely.
7. **Analysis reporting bias** examined whether the study was free from selective analysis reporting, for example by ensuring the authors report results corresponding to the outcomes announced in the method section.

The included studies were coded as 'critical risk', 'high risk', medium risk' or 'low risk' across these domains and a final score was given as follows:

- Studies were assigned as having an overall critical risk of bias if they suffered from a critical risk of bias in the confounding domain.
- Studies were assigned as having an overall high risk of bias if they had one or more domains marked as having a high risk of bias, regardless of whether all other domains were scored as medium or low risk.
- Studies were assigned as having an overall medium risk of bias if they had one or more domains marked as having a medium risk of bias, regardless of whether all other domains were scored as low risk.
- Studies were assigned as having an overall low risk of bias if they had all domains marked as having a low risk of bias.
- Studies where the confounding domain could not be scored due to insufficient information were marked as 'high-risk' for this domain. All other domains were marked as 'medium score' if there was insufficient information to give a score.

Figure F1: Overall risk of bias

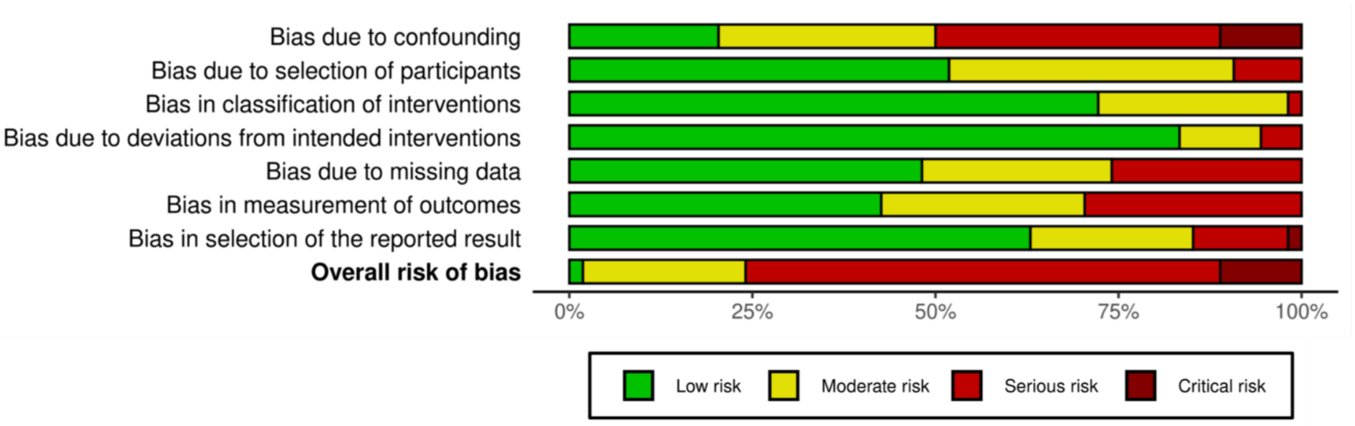

Figure F2: Study level risk of bias (See Appendix G for Author, date information)

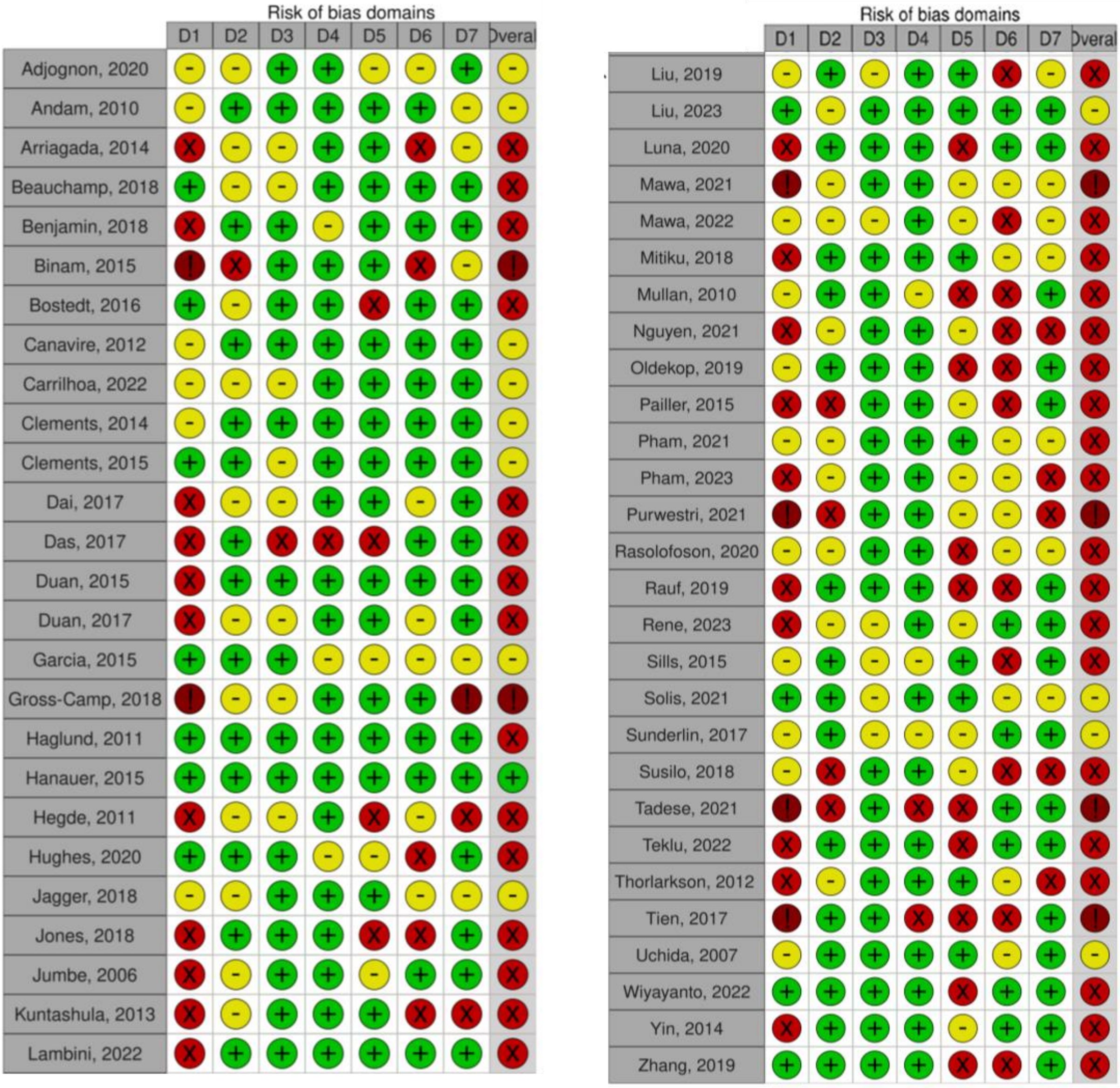

Domains
D1: Confounding bias
D2: Selection bias
D3: Attrition bias
D4: Motivation bias
D5: Performance bias
D6: Measurement and reporting error
D7: Analysis reporting bias

Assessment
Critical
High
Medium
Low

Appendix G: List of included studies summarised by type of intervention, implementation country, study design and risk of bias assessment summary score

	Author, year	Intervention in brief:	Country and Region	Scale (unit of analysis, sampled from)	Study design and methods of analysis	Risk of bias assessment
1.	Adjognon, 2020[45]	Payment for Environment Services (PES) under the government Forest Investment Program (FIP)	Burkina Faso, Africa	Households, local areas	Randomized Controlled Trial with adjusted regression estimation	Medium risk
2.	Andam, 2010[70]	Protected Areas (PA)	Thailand and Costa Rica (Asia and Central America)	Household, districts	Matching estimator and adjusted linear regression applied to longitudinal data	Medium risk
3.	Arriagada, 2014[72]	Payment for Ecosystem Services (PES)	Costa Rica, Central America	Household, cantons	Matching estimator and adjusted regression applied to cross-section data with recalled baseline	High risk
4.	Beauchamp, 2018[22]	Protected Areas and 3 PES interventions (bird nest, ecotourism and Ibis rice)	Cambodia, Asia	Households, villages	Propensity score matching (PSM) and difference in differences (DiD) regression applied to longitudinal data	High risk
5.	Benjamin, 2018[76]	Agroforestry schemes with payment for ecosystem services (PES)	Kenya, Africa	Households, counties	Adjusted ordinary least squares (OLS) regression applied to cross-section data	High risk
6.	Binam, 2015[16]	Farmer Managed Natural Regeneration (FMNR)	Burkina Faso, Mali, Niger and Senegal (Sahel, Africa)	Households, villages	Inverse propensity weighted regression applied to cross-section data	Critical risk
7.	Bostedt, 2016[49]	Agroforestry, in agro-pastoral communities	Kenya, Africa	Households, local areas	Heckman two-stage regression applied to cross-section data	High risk
8.	Canavire-Bacarreza, 2012[69]	Protected Areas (PA)	Bolivia, South America	Municipalities	Genetic matching with post-match regression bias adjustment applied to longitudinal data	Medium risk
9.	Carrilhoa, 2022[81]	Reducing Emissions from Deforestation and Forest Degradation (REDD+)	Brazil, South America	Households, local areas	DiD regression estimation applied to longitudinal data	Medium risk
10.	Clements, 2014[24]	Protected Areas	Cambodia, Asia	Households, villages	Double-matching of villages and households within villages, using mixed effect models applied to cross-section data	Medium risk
11.	Clements, 2015[10]	Protected Areas and 3 PES interventions (bird nest, ecotourism, and Ibis rice)	Cambodia, Asia	Households, villages	DiD regression estimation applied to longitudinal data	Medium risk

12.	Dai, 2017[52]	Agroforestry	China, Asia	Households, villages	PSM with comparison of means applied to cross-section data	High risk
13.	Das, 2017[43]	Mangrove restoration with enriched and natural trees	India, Asia	Households, villages	DiD applied to longitudinal data	High risk
14.	Duan, 2015[58]	Sloping Land Conversion Program, one of the world's largest payment for ecosystem services world (PES)	China, Asia	Households, villages	Adjusted OLS, Tobit and quantile regression estimation applied to cross-section data	High risk
15.	Duan, 2017[55]	Protected Areas (PA)	China, Asia	Households, villages	Matching estimator and Tobit regression estimation applied on cross-section data	High risk
16.	Garcia and Sims, 2015[11]	Payments for Hydrological Services – a type of PES	Mexico, Central America	Households, local areas (ejidos)	Matching estimators and DiD estimation applied to longitudinal data	Medium risk
17.	Gross-Camp, 2017[46]	Collaborative Forest Management Community-based, participatory forest management (CBFM) compared to centralized forest management	Tanzania, Africa	Households, villages	Matching estimator and mixed effects models applied to cross-section data	Critical risk
18.	Haglund, 2011[17]	Farmer Managed Natural Regeneration - Dry land tree management (FMNR)	Niger, Africa	Households, villages	PSM with comparison of means applied to cross-section data	High risk
19.	Hanauer, 2015[7]	Protected Areas (PA)	Bolivia, South America	Cantons	Post-matching regression estimation, applied to longitudinal data	Low risk
20.	Hegde, 2011[51]	Agroforestry based Payment for Ecosystem Services (PES)	Mozambique, Africa	Households, villages	PSM with comparison of means applied to cross-section data	High risk
21.	Hughes, 2020[39]	Agroforestry	Kenya, Africa	Households, villages	DiD and instrumental variable (IV) estimation applied to longitudinal data	High risk
22.	Jagger, 2018[67]	Collaborative Forest Management using management agreements	Uganda, Africa	Households, villages	DiD regression estimation applied to longitudinal data	Medium risk
23.	Jones, 2018[73]	Payments for hydrological services (PHS) a type of PES	Mexico, North America	Households, local areas (ejidos)	DiD regression estimation applied to longitudinal data	High risk
24.	Jumbe, 2006[65]	Collaborative Forest Management using a forest co-management program	Malawi, Africa	Households, villages	PSM with comparison of means applied to cross-section data	High risk
25.	Kuntashula, 2013[41]	Agroforestry using improved fallows	Zambia, Africa	Households, villages	PSM and endogenous switching regression (ESR) applied to cross-section data	High risk
26.	Lambini, 2022[68]	Collaborative Forest Management using community-based conservation	Kenya, Africa	Households, local areas	PSM with comparison of means applied to cross section data	High risk
27.	Liu, 2019[77]	Collaborative Forest Management	China, Asia	Households, sub-regional	Adjusted OLS regression applied to longitudinal data	High risk

28.	Liu, 2023[79]	Forest Farm Carbon Sink (FFCS) to promote carbon mitigation and increase environmental and ecological benefits	China, Asia	County	PSM with DiD regression estimation applied to longitudinal data with pre-test	Medium risk
29.	Luna, 2020[6]	Protected areas (PA) and PES under Socio Bosque incentive based conservation program	Ecuador, South America	Households, local areas	Tobit regression applied to cross-sectional data	High risk
30.	Mawa, 2021[75]	Collaborative Forest Management	Uganda, Africa	Households, villages	PSM applied to cross-section data with comparison of means	Critical risk
31.	Mawa, 2022[66]	Collaborative Forest Management	Uganda, Africa	Households, villages	PSM applied to cross-section data with comparison of means	High risk
32.	Mitiku, 2018[9]	Agroforestry based on coffee semi-forests	Ethiopia, Africa	Households, districts	Adjusted OLS and fixed effects regression applied to cross-sectional data	High risk
33.	Mullan, 2010[63]	Natural Forest Protection Program (NFPP), national level forests protection and afforestation initiative	China, Asia	Households, counties	PSM and DiD regression estimation applied to longitudinal data	High risk
34.	Nguyen, 2021[61]	Payments for Environmental Services (PES)	Vietnam, Asia	Households, communes	PSM with comparison of means applied to cross-section data	High risk
35.	Oldekop, 2019[71]	Community-based Forest Management	Nepal, Asia	Sub-districts, national	Matching estimator and fixed effects regression applied to longitudinal data	High risk
36.	Pailler, 2015[47]	Collaborative Forest Management using a community-based natural resource management (CBNRM)	Tanzania, Africa	Villages, national	DiD applied to longitudinal data	High risk
37.	Pham, 2021[57]	State-run PES	Vietnam, Asia	Households, villages	PSM with comparison of means applied to	High risk
38.	Pham, 2023[56]	Payment for Ecosystem Services (PES)	Vietnam, Asia	Households, villages	PSM with comparison of means applied to cross-section data	High risk
39.	Purwestri, 2021[48]	Agroforestry	Indonesia, Asia	Households, sub-districts	Binary logistic regression estimation applied to cross-sectional data	Critical risk
40.	Rasolofoson, 2017[86]	Collaborative Forest Management	Madagascar, Africa	Households, national survey data	Matching estimator and adjusted regression estimation applied to cross-section data	High risk
41.	Rauf, 2019[62]	National Forest Protection Program under the Billion Trees Afforestation Program (BTAP) the largest afforestation program in Pakistan	Pakistan, Asia	Households, villages	Ordered logit model and ordinary least squares (OLS) estimation applied to cross-section data	High risk

42.	Rene, 2023[40]	Certified cocoa-based agroforestry systems	Cameroon, Africa	Households, divisions	Propensity Score Matching (PSM) and endogenous switching regression (ESR) applied to cross-section data	High risk
43.	Sills, 2015[50]	Agroforestry and conservation agriculture to reduce cattle ranching to minimise deforestation.	Brazil, South America	Households, municipalities	Matching estimators and adjusted OLS regression applied to cross-section data	High risk
44.	Solis, 2021[78]	REDD+	Peru, South America	Households, local area	DiD estimation applied to longitudinal data	Medium risk
45.	Sunderlin, 2017[53]	REDD+	Brazil, Peru, Cameroon, Tanzania, Indonesia, Vietnam	Households, villages	DiD estimation applied to longitudinal data with pre-test	Medium risk
46.	Susilo, 2018[64]	Integrated mangrove–shrimp farming	Indonesia, Asia	Households, villages	PSM and logistic regression applied to cross-section data	High risk
47.	Tadesse, 2021[42]	Agroforestry based on enset growing	Ethiopia, Africa	Households, district	Linear mixed model applied to cross-section data	Critical risk
48.	Teklu, 2022[44]	Agroforestry	Ethiopia, Africa	Households, woreda	Endogenous switching regression (ESR) and instrumental variables (IV) applied to cross-section data	High risk
49.	Thorlakson, 2012[74]	Agroforestry	Kenya, Africa	Households, sub-district	Matching estimators and linear regression applied to cross-section data	High risk
50.	Tien, 2017[54]	REDD+	Vietnam, Asia	Households, communes	Naïve comparison of means using ttest applied to cross-section data	Critical risk
51.	Uchida, 2007[59]	Sloping Land Conversion Program, one of the world's largest payment for ecosystem services world (PES)	China, Asia	Households, villages	PSM and DiD regression estimation applied to longitudinal data	Medium risk
52.	Wijayonto, 2022[80]	Agroforestry	Indonesia, Asia	Households, sub-district	PSM with comparison of means applied to cross-section data	High risk
53.	Yin, 2014[60]	Sloping Land Conversion Program, one of the world's largest payment for ecosystem services world (PES)	China, Asia	Households, villages	Fixed effects regression applied to longitudinal data with recalled baseline	High risk
54.	Zhang, 2019[12]	State-initiated PES: Sloping Land Conversion Program and the Ecological Welfare Forest Program (EWFP)	China, Asia	Households, villages	Mixed effects models applied to cross-sectional data	High risk

Appendix H: Models without adjustment for effect dependence

Outcome	Overall effects (95%CI), p-value	I-statistic	T-statistic	Q-statistic
Agricultural yields	0.33 (0.11-0.55), p=0.003	94.2%	0.25	263.42
Food security	0.10 (0.01, 0.18), p=0.025	80.9%	0.02	61.90
Dietary Diversity *(same as adjusted effects)*	0.10 (0.02, 0.18), p=0.012	0%	0	1.72
Total income	0.16 (0.07, 0.25), p<0.001	86.9%	0.06	312.71
Income from agricultural production	-0.02 (-0.12, 0.08), p=0.745	72.6%	0.03	49.79
Income from non-timber forest products	0.27 (0.09, 0.45), p=0.004	0%	0.10	88.00
Income from timber products *(same as adjusted effects)*	-0.13 (-0.29, -0.02), p<0.093	37.5%	0.01	3.4
Poverty reduction	0.17 (0.07, 0.27), p<0.001	76.5%	0.03	47.54
Self-reported wellbeing	0.13 (0.01-0.26), p=0.034	81.2%	0.04	53.97
Child growth	0.14 (0.07-0.22), p<0.001	84.5%	0.01	49.71
Access to education	0.51 (-0.13, 1.16), p=0.118	93%	0.4	51.79

Appendix I: Funnel plots and Egger's regression test for funnel plot asymmetry (conducted where studies >=10)

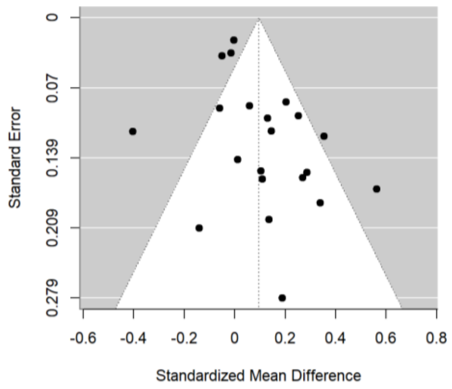

Food Security
z = 1.7714, p = 0.0765

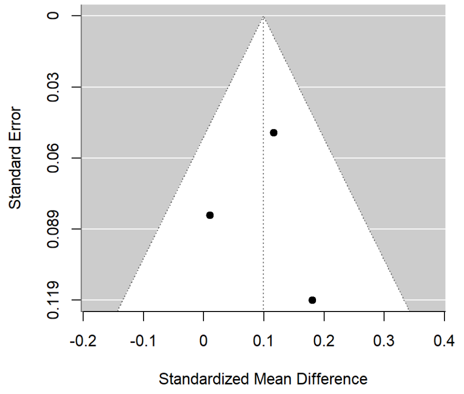

Dietary diversity

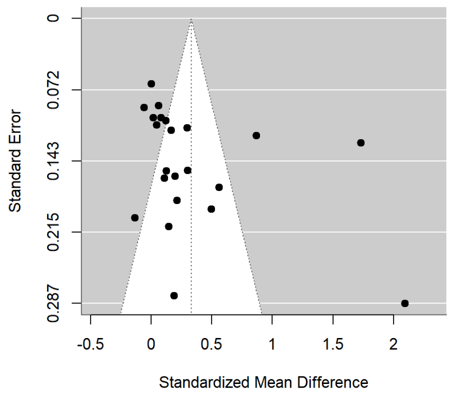

Yields
z = 1.7960, p = 0.0725

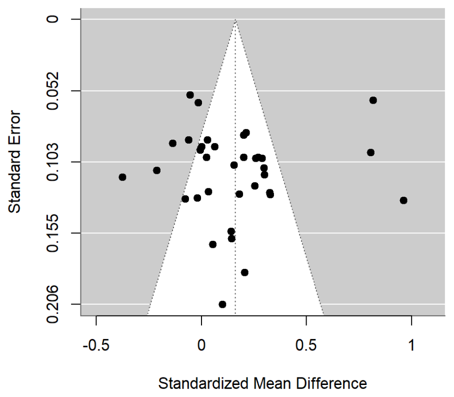

Income - Total
z = -0.0638, p = 0.9491

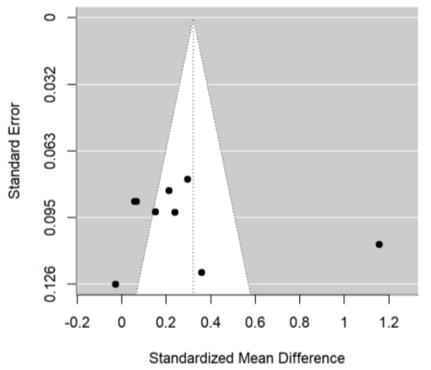

Income – Non-timber

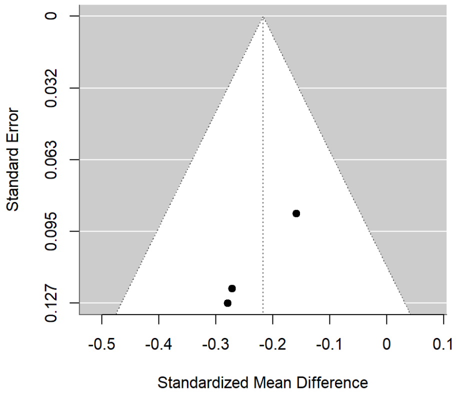

Income – Timber

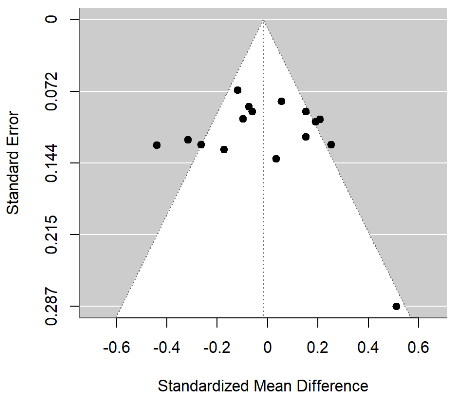

Income – Agriculture
z = 0.8851, p = 0.3761

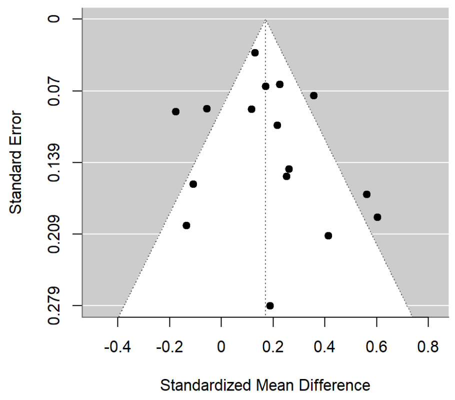

Poverty
z = 0.8966, p = 0.3699

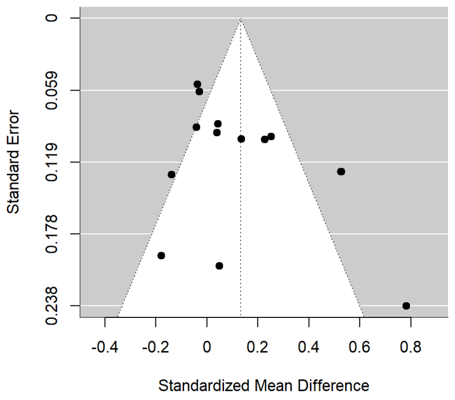

Wellbeing
z = 1.5091, p = 0.1313

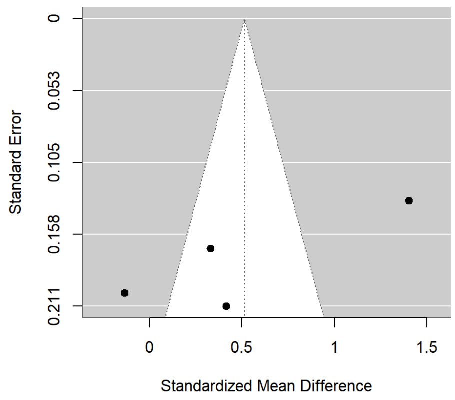

Education

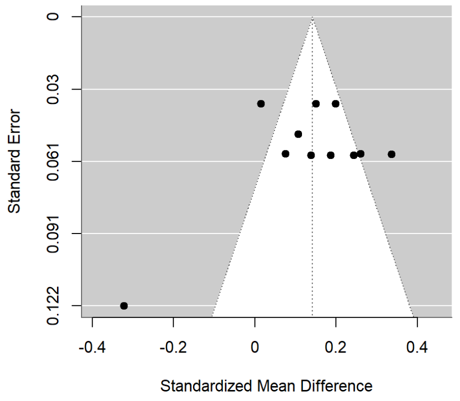

Child growth
